# Economic evaluation of HPV DNA test as primary screening method for cervical cancer: A health policy discussion in Greece

**DOI:** 10.1371/journal.pone.0226335

**Published:** 2019-12-12

**Authors:** Anastasios Skroumpelos, Theodoros Agorastos, Theodoros Constantinidis, Kimon Chatzistamatiou, John Kyriopoulos

**Affiliations:** 1 Roche Pharmaceuticals (Hellas) S.A., Athens, Greece; 2 Medical School, Aristotle University, Thessaloniki, Greece; 3 Medical School, Dimocrition University, Komotini, Greece; 4 Department of Health Economics, National School of Public Health, Athens, Greece; Universitat Bremen, GERMANY

## Abstract

**Background:**

HPV test appears to be more effective in cervical cancer (CC) screening. However, the decision of its adoption as a primary screening method by substituting the established cytology lies in the evaluation of multiple criteria. Aim of this study is to evaluate the economic and clinical impact of HPV test as primary screening method for CC.

**Methods:**

A decision tree and a Markov model were developed to simulate the screening algorithm and the natural history of CC. Fourteen different screening strategies were evaluated, for women 25–65 years old. Clinical inputs were drawn from the HERMES study and cost inputs from the official price lists. In the absence of CC treatment cost data, the respective Spanish costs were used after being converted to 2017 Greek values. One-way and probabilistic sensitivity analyses were conducted.

**Results:**

All screening strategies, that offer as primary screening method triennial HPV genotyping (simultaneous or reflex) alone or as co-testing with cytology appear to be more effective than all other strategies, with regards to both annual CC mortality, due to missed disease (-10.1), and CC incidence(-7.5) versus annual cytology (current practice). Of those, the strategy with HPV test with simultaneous 16/18 genotyping is the strategy that provides savings of 1.050 million euros annually. However, when the above strategy is offered quinquennially despite the fact that outcomes are decreased it remains more effective than current practice (-7.7 deaths and -1.3 incidence) and more savings per death averted (1.323 million) or incidence reduced (7.837 million) are realized.

**Conclusions:**

HPV 16/18 genotyping as a primary screening method for CC appears to be one of the most effective strategies and dominates current practice in respect to both cost and outcomes. Even when compared with all other strategies, the outcomes that it generates justify the cost that it requires, representing a good value for money alternative.

## Introduction

Cervical cancer represents the fourth most frequent cancer in women worldwide and the eighth in Europe, with annual new incidence of 569,847 and 61,072, respectively [1]. The burden of disease in Greece is estimated at 696 incidence [1] annually and cervical cancer is responsible for 21.6 potential years of life lost / 100,000 females [2] and 5,800 disability adjusted life years [3].

Since its introduction, in 1954 [4], and its adoption as a screening method, the Papanicolaou test (Pap test) has contributed considerably in the prevention of cervical cancer in the developed countries but less in low income countries due to the absence of effective national policies and low compliance rates [5]. Until a few years ago, cytology was the primary method in all the developed health care systems for the detection and prevention of cervical cancer. In Greece Pap-test is still the primary screening method offered annually for all women and fully covered by the social insurance. However, there is not an organized screening program and only 30.3% of women appear to perform the test regularly and annually for more than 5 years [6].

In the late 1970s, the human papillomavirus (HPV) was associated with the development of squamous cell carcinomas [7] and is currently perceived as the main risk factor for invasive cervical cancer (CC) [8]. Twelve types of the virus (16, 18, 31, 33, 35, 39, 45, 51, 52, 56, 58, and 59) have been proven to be high-risk regarding carcinogenicity [8] and, out of these, types 16 and 18 are considered as those with the highest risk. Hence, this can explain the fact that 76.2% of the cervical cancers in Europe have been explicitly attributed to genotypes 16 and 18 [9].

The association of HPV with cervical cancer led to the development of diagnostic methods for the detection of the virus’s DNA (HPV DNA test), which can ultimately be used as a primary screening method for cervical cancer screening and substitute or complement the Papanicolaou test. The performance of the HPV DNA test has been proven superior to the Pap test in the detection of CIN2 or greater, as the latter has demonstrated sensitivity that varies from 44% to 74%, with an average of 53%, while the sensitivity of the HPV DNA test reaches 100% [10–16]. The specificity of the HPV DNA test appears slightly lower than Pap’s test as it has been estimated at 90.3% versus 96.8% of the cytology’s [12].

However, according to Cochrane and Holland [17, 18] beyond a diagnostics method’s performance (sensitivity and specificity), several additional criteria should be fulfilled in order for the intervention to be adopted as a method for population screening. These are the intervention’s a) simplicity as it should be easy to be performed and interpreted and capable of use even by paramedics and other personnel, b) acceptability by the individuals, c) accuracy, as it should provide with a true measurement of the condition or symptom under investigation, d) repeatability, as it should provide consistent results in repeated trials and last but not least e) cost.

The aim of the study is the investigation of the latter by exploring the economic efficiency of the HPV test as a primary screening method for cervical cancer in Greece. The cobas HPV test was used for the current analysis which detects 14 high-risk types (16, 18, 31, 33, 35, 39, 45, 51, 52, 56, 58, 59, 66, 68) and simultaneously the 16 and the 18 genotypes. The study is conducted from the social insurance’s perspective and compares the budget impact, the cost per death averted and the cost per incidence reduced of screening with a) cytology annually (current practice) and triennially, b) the HPV test with simultaneous HPV 16 and 18 genotyping every three and every five years, c) the HPV test with reflex 16 and 18 genotyping every three and every five years, d) the HPV test with no genotyping every three and every five years, e) Co-testing with cytology and the HPV test with simultaneous 16 and 18 genotyping every three and every five years, f) Co-testing with cytology and the HPV test with reflex 16 and 18 genotyping every three and every five years, g) Co-testing with cytology and the HPV test with no genotyping every three and every five years. The aim of the study was achieved by providing a comparative evaluation of both the clinical and economic impact of all the above screening strategies, under two reimbursement scenarios.

## Materials and methods

A decision tree was developed to model the screening and diagnosis of cervical cancer. The baseline assumption of individuals’ compliance to cervical cancer screening was 30.3% [6]. The model timelines are equivalent to two screening intervals, according to each strategy’s routine screening interval. The performance of the test and the incidence of the disease determine whether women are sent to follow-up testing or routine screening. All screened women between the ages of 25 to 65 enter the model at the same time and exit the model when they are indicated for treatment (either diagnosed with CIN2, CIN3 or invasive cervical cancer). Women may be lost to follow-up at re-test or at the next routine screening interval, at which time they may become infected with HPV or their disease can either progress or regress. Between screening intervals, women may become infected with HPV or their disease can either progress or regress. The natural history of HPV and cervical cancer is simulated by a Markov model.

**Fig 1:**
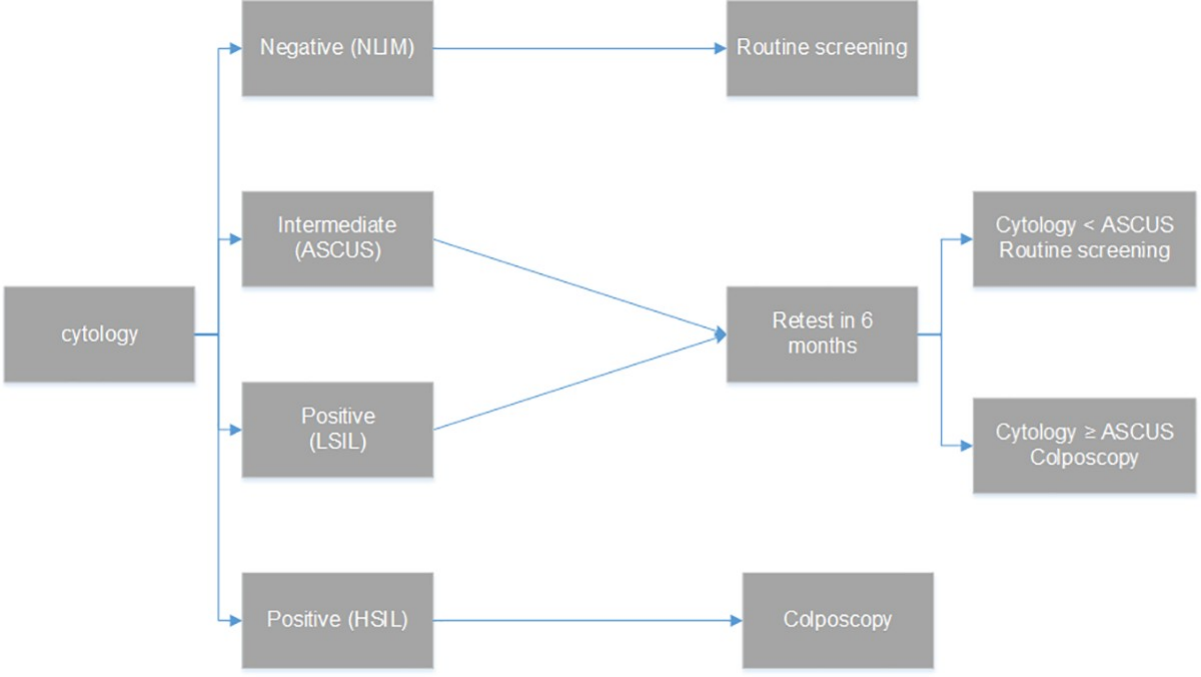
Cytology screening strategy.

**Fig 2:**
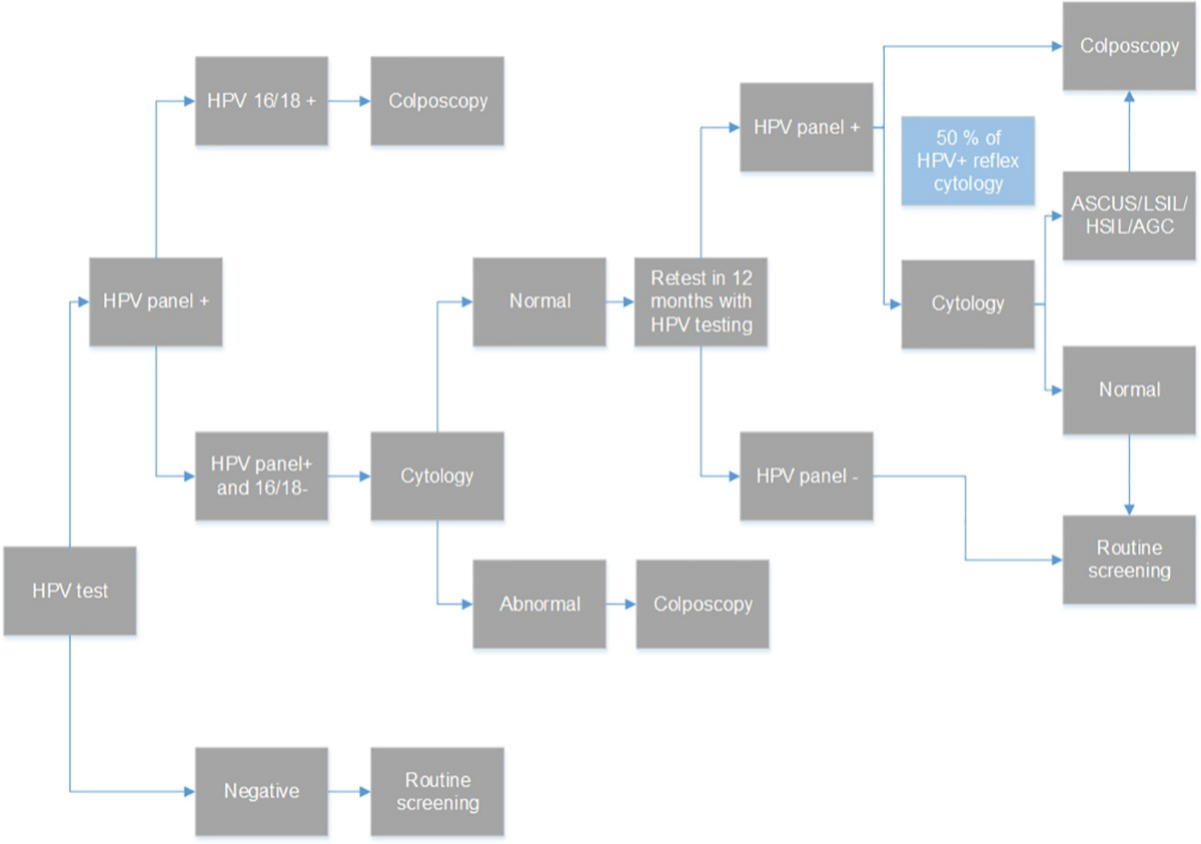
Primary HPV test with 16/18 genotyping screening strategy.

**Fig 3:**
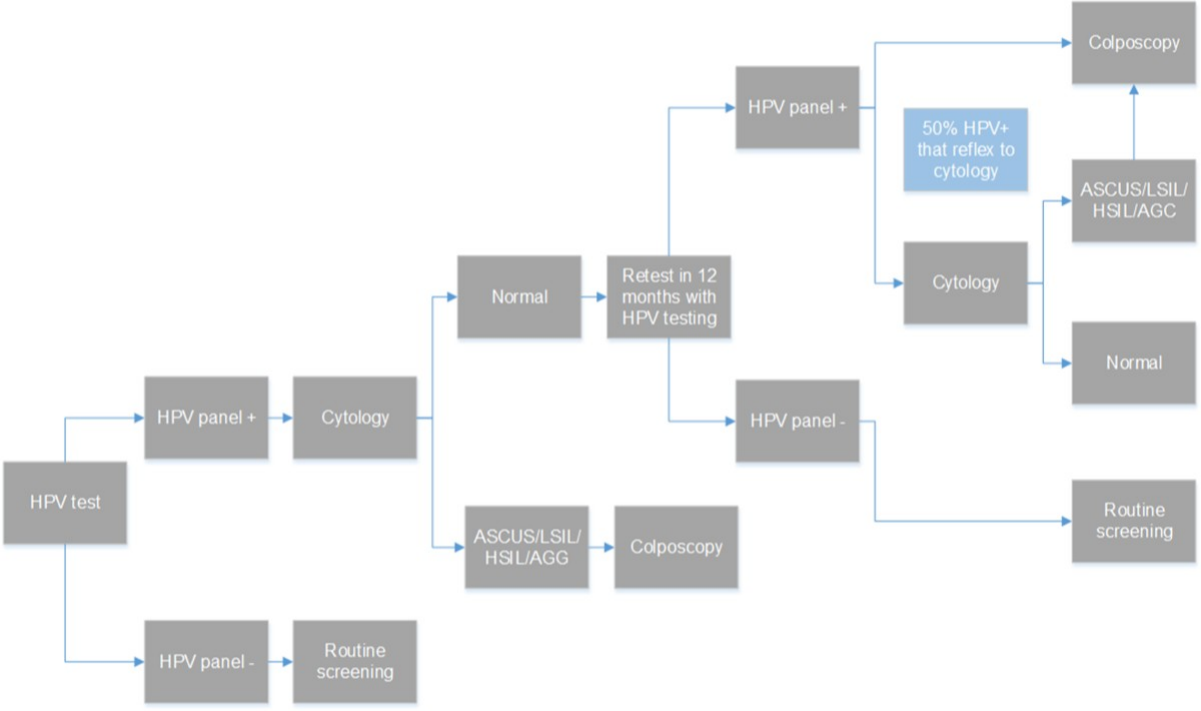
Primary HPV test with no genotyping screening strategy.

**Fig 4:**
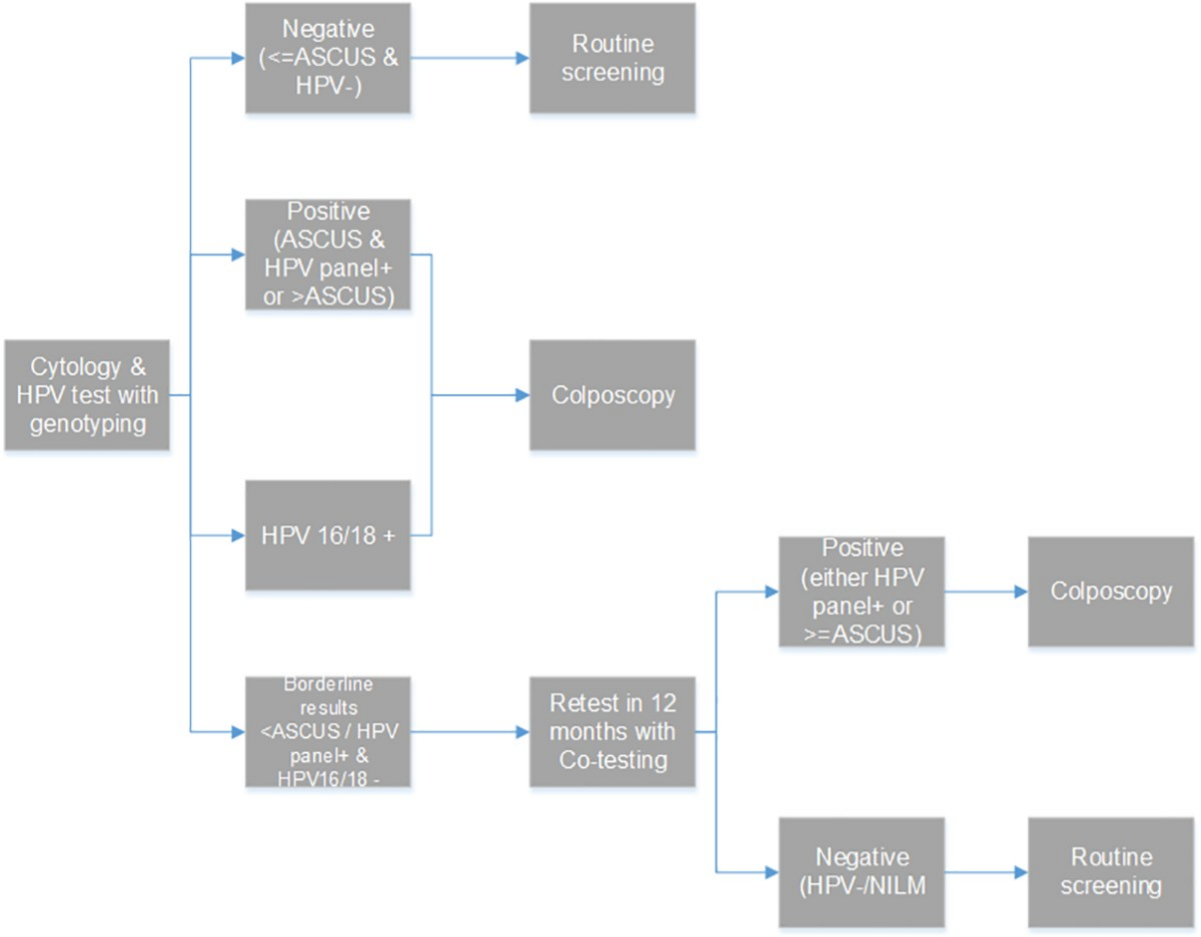
Co-testing with cytology and HPV with genotyping screening strategy.

**Fig 5:**
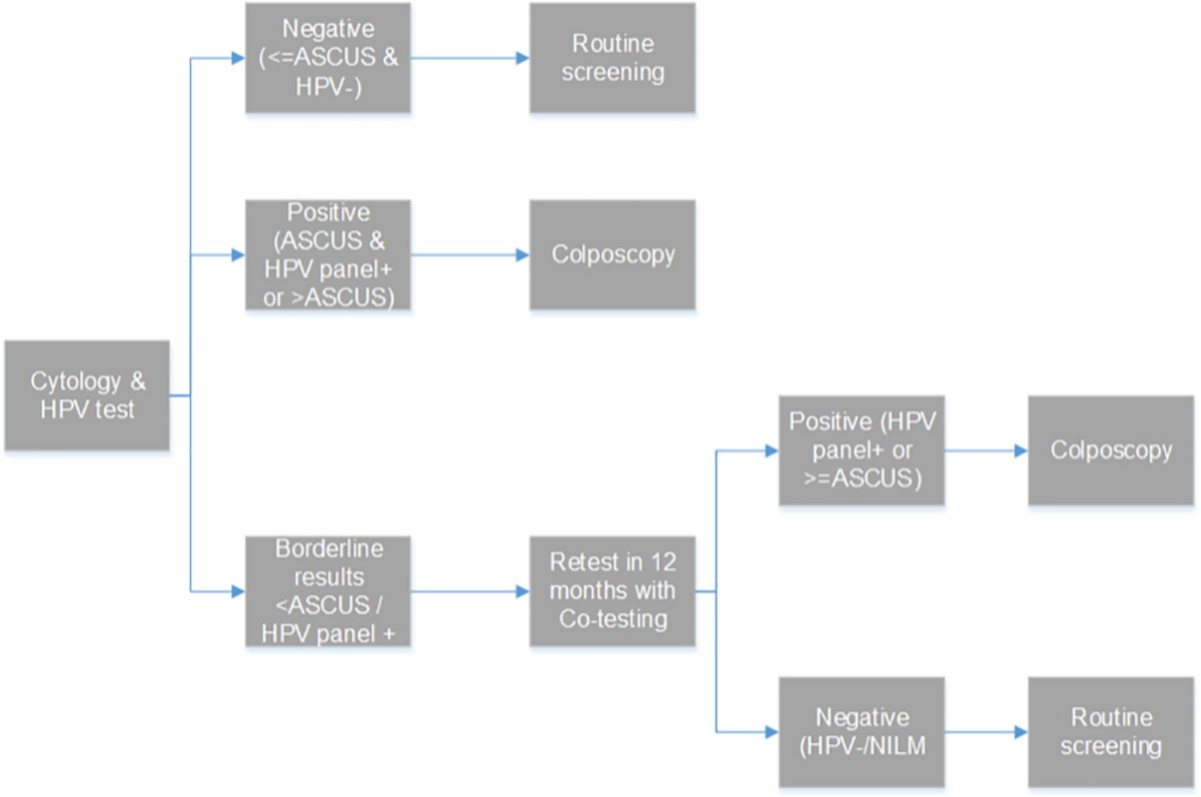
Co-testing with cytology and HPV with no genotyping screening strategy.

The compared screening algorithms and strategies are:

a) Cytology: Cytology is offered and reimbursed annually by the social insurance fund in Greece. The model’s algorithm determines that a woman with negative cytology results returns to routine screening in one or three years. Women with intermediate results—referred to as atypical squamous cells of undetermined significance (ASCUS)—are re-tested with cytology in 6 months, and then, in case of cytology results of ASCUS or worse they are referred to immediate colposcopy, otherwise they return to routine screening. In case of positive results at the primary test [Low Grade Squamous Intraepithelial Lesion(LSIL), High Grade Squamous Intraepithelial Lesion (HSIL), Atypical Glandular Cells (AGC)] women are referred to colposcopy (Fig 1).

b) Primary HPV test with 16/18 genotyping: HPV screening is assumed to be offered every 3 or 5 years. Women with negative results return for routine screening in 3 or 5 years. Women who are HPV 16/18 positive are referred for immediate colposcopy. Women who are HPV 16/18 negative, but positive to the rest of the 12 high risk HPV types are referred to cytology. In case of abnormal results, women are referred to colposcopy. Normal results are retested in 12 months and half of them that test hrHPV positive receive cytology and the rest colposcopy (Fig 2).

c) Primary HPV test with no genotyping: HPV screening is provided every 3 or 5 years. As above women with no HPV findings return to routine screening. HPV positive women are referred for cytology and those with negative results are retested with HPV test in 12 months. Women with intermediate (ASCUS) and or positive results (LSIL, HSIL, AGC) are referred for colposcopy (Fig 3).

d) Co-testing with cytology and HPV with genotyping. Both cytology and HPV test are offered every 3 or 5 years. Women with normal cytology results who are HPV negative return to routine screening. Those with positive HPV results and/or ASCUS cytology results and those positive to 16/18 genotypes are referred for colposcopy. Women with borderline cytology results (<ASCUS) and positive to HPV but negative to 16/18 are retested in 12 months (Fig 4).

e) Co-testing with cytology and HPV with no genotyping. Both tests are offered every 3 or 5 years. As above, women with negative cytology and HPV results return to routine screening. Those with cytology results worse than ASCUS or ASCUS and HPV positive are referred for colposcopy. In the case of borderline results (<ASCUS) and HPV positive are retested in 12 months with co-testing (Fig 5).

**Fig 6:**
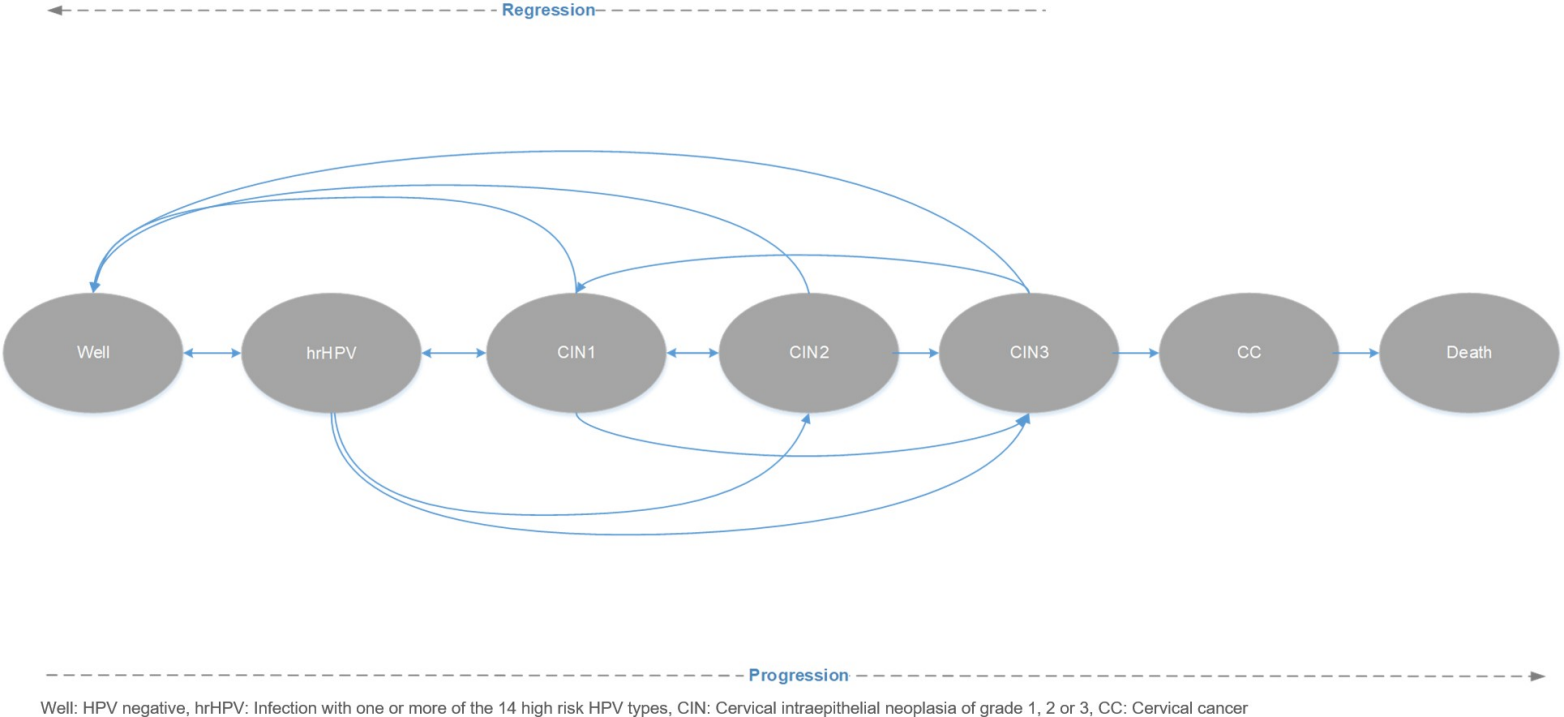
Natural history model of cervical cancer.

As mentioned above a Markov 7-state transition model with 1-month cycle (inputs are entered annually and converted to monthly) was developed to simulate women’s transition from one state of the disease to another (Fig 6). Women may transition to different health states when they are (1) lost to follow-up or (2) between screening intervals. The health states include Well, HPV16/18, hrHPV (12 pooled), cervical intraepithelial neoplasia of mild degree (CIN1), CIN2, CIN3, CC and Death (Fig 6). The model does not differentiate between the different stages of CC and the average probabilities of progression and regression of CIN used were not stratified by HPV type. The model only includes the probability of dying from CC and all-cause mortality is not considered. The annual probability of progression and regression to each state is based on published literature. The risk of progression and regression was simplified to be constant over time and is not stratified by age. The main clinical outcomes that the model estimates are annual cervical cancer incidence and cervical cancer deaths. All outcomes were discounted with a rate of 3.5%.

The vast majority of clinical, epidemiological and diagnostic tests’ performance inputs were drawn from the HERMES study (HEllenic Real life Multicentric cErvical Screening) [12], which was carried out in Greece from April 2011 until September 2013 and recruited 4,009 women (Table 1). The respective study used the cobas HPV test and thus, the characteristics of the specific test were used in the current analysis. In addition, it was assumed that the clinical outcomes regarding the detection of all the high risk genotypes do not differ significantly from other HPV panel tests. Colposcopy’s sensitivity and specificity was assumed to be 100%. Due to the fact that the HERMES study did not detect any CC case, the sensitivity of all diagnostic tests for the detection of cancer was assumed to be equal to that for the detection of CIN3. The Markov’s model transition probabilities for the simulation of the natural history of the disease were drawn from international literature. In the case of multiple sources, a weighted average was used (Table 2).

**Table 1:** Clinical data.

	Base case	Range	References
Screened population			
Total population	10,816,286	-	Hellenic Statistical Authority (2017) [19]
% of females between the age 25 and 65	27,7%	-	Hellenic Statistical Authority (2017) [19]
*Test performance ≥ CIN 2*			
*Cytology (threshold = ASCUS)*			
% of population testing ASCUS or worse	5.3%		Agorastos et al. (2015) [12]
Sensitivity of cytology for CIN 2+	53.7%	37.4–69.3	Agorastos et al. (2015) [12]
Sensitivity of cytology for CIN 3+	64.3%	35.1–87.2	Agorastos et al. (2015) [12]
Sensitivity of cytology for CC	64.3%*	35.1–87.2	Agorastos et al. (2015) [12]
Specificity of cytology	96.8	96.2–97.4	Agorastos et al. (2015) [12]
*Cytology (threshold = LSIL)*			
% of population testing LSIL	1.9%		Agorastos et al. (2015) [12]
Sensitivity of cytology for CIN 2+	41.5%	26.3–57.9	Agorastos et al. (2015) [12]
Sensitivity of cytology for CIN 3+	57.1%	28.9–82.3	Agorastos et al. (2015) [12]
Sensitivity of cytology for CC	57.1%*	28.9–82.3	Agorastos et al. (2015) [12]
Specificity of cytology	98.8	98.4–99.1	Agorastos et al. (2015) [12]
*Cytology (threshold = HSIL)*			
% of population testing HSIL	0.4%		Agorastos et al. (2015) [12]
Sensitivity of cytology for CIN 2+	17.7%	8.5–31.3	Agorastos et al. (2015) [12]
Sensitivity of cytology for CIN 3+	21.43%	7.6–47.6	Agorastos et al. (2015) [12]
Sensitivity of cytology for CC	21.43%	7.6–47.6	Agorastos et al. (2015) [12]
Specificity of cytology	99.8%	99.6–99.9	Agorastos et al. (2015) [12]
*HPV testing*			
% of population that is cytology+ HPV+	2.8%		Agorastos et al. (2015) [12]
Sensitivity of HPV test for CIN2	100%	91.4–100.0	Agorastos et al. (2015) [12]
Sensitivity of HPV test for CIN3	100%	76.8–100.0	Agorastos et al. (2015) [12]
Sensitivity of HPV test for CC	100%	76.8–100.0	Agorastos et al. (2015) [12]
Specificity of HPV testing	90.3%	89.3–91.2	Agorastos et al. (2015) [12]
Sensitivity of HPV test with 16/18 genotyping for CIN2	58.5%	42.1–73.7	Agorastos et al. (2015) [12]
Sensitivity of HPV test with 16/18 genotyping for CIN3	78.6%	49.2–95.3	Agorastos et al. (2015) [12]
Sensitivity of HPV test with 16/18 genotyping for CC	78.6%	49.2–95.3	Agorastos et al. (2015) [12]
Specificity of HPV test with 16/18 genotyping	97,5%	96.9–98.0	Agorastos et al. (2015) [12]
*Colposcopy*			
Sensitivity of colposcopy for CIN1	100%	-	Model’s assumption
Sensitivity of colposcopy for CIN2	100%	-	Model’s assumption
Sensitivity of colposcopy for CIN3	100%	-	Model’s assumption
Sensitivity of colposcopy for CC	100%	-	Model’s assumption
Specificity of colposcopy	100%		Model’s assumption

* No CC cases were observed in the HERMES study thus, the respective sensitivity for CIN3 was used

**Table 2:** Natural history parameters.

	Base case	References
*Natural history parameters*		
Well to hrHPV	4.2%	Kulasingam SL, et al. (2013)[20]
Progression from hrHPV (12)*		
to CIN1	8.1%	Kulasingam et al. (2013) [20], Kjær et al. (2010) [21]
to CIN2	0.1%	Khan et al. (2005) [22]
to CIN3	0.1%	Khan et al. (2005) [22]
Progression from hrHPV 16/18		
to CIN1	9.9%	Kjær et al. (2010) [21], Khan et al. (2005)[22], Insinga et al. (2007) [23], Insinga et al. (2011) [24]
to CIN2	0.6%	Kjær et al. (2010) [21], Khan et al. (2005) [22], Insinga et al. (2007) [23], Insinga et al. (2011) [24]
to CIN3	1.5%	Kjær et al. (2010) [21], Khan et al. (2005)[22], Insinga et al. (2007) [23], Insinga et al. (2011) [24]
Progression from CIN1		
to CIN2	3.2%	Kataja et al. (1989) [25], Holowaty et al. (1999) [26] Matsumoto et al. (2006) [27]
to CIN3	0.9%	Kataja et al. (1989) [25], Holowaty et al. (1999)[26]
Progression from CIN2		
to CIN3	4.2%	Kataja et al. (1989) [25], Holowaty et al. (1999) [26], Matsumoto et al. (2006) [27], Guedes et al. (2010) [28], Omori et al. (2007) [29]
to CC	0.0%	base case assumes CIN2 does not progress directly to CC
Progression from CIN3		
to CC	1.1%	Kulasingam et al. (2013) [20], Kataja et al. (1989) [25], Holowaty et al. (1999) [26], McCredie et al. (2008) [30], Sasieni et al. (2009) [31], Goldie et al. (2004) [32], Mandelblatt et al. (2002) [33], Insinga et al. (2009) [34]
Progression from CC		
to death	0.6%	National Cancer Institute [35]
Regression from hrHPV (12)		
Normal smear to well	58.6%	Bulkmans et al. (2007) [36]
Abnormal smear to well	45.6%	Bulkmans et al. (2007) [36]
Regression from hrHPV 16/18		
Normal smear to well	43.8%	Insinga et al. (2011) [24], Bulkmans et al. (2007)[36]
Abnormal smear to well	21.8%	Insinga et al. (2011) [24], Bulkmans et al. (2007)[36]
Regression from CIN1		
to well	21.2%	Kataja et al. (1989) [25], Holowaty et al. (1999) [26], Matsumoto et al. (2006) [27]
to hrHPV (12)	2.4%	Kataja et al. (1989) [25], Holowaty et al. (1999) [26], Matsumoto et al. (2006) [27]
Regression from CIN2		
to well	9.4%	Kataja et al. (1989) [25], Holowaty et al. (1999) [26], Guedes et al. (2010) [28], Omori et al. (2007) [29]
to CIN1	9.4%	Kataja et al. (1989) [25], Holowaty et al. (1999) [26], Guedes et al. (2010) [28], Omori et al. (2007) [29], Meyskens et al. (1994)[37], Castle et al. (2009) [38]
Regression from CIN3		
to well	3.9%	Kataja et al. (1989) [25], McCredie et al. (2008) [30]
to CIN1	1.6%	Kataja et al. (1989) [25], McCredie et al. (2008) [30]
*Epidemiology parameters*		
Prevalence of 14hrHPV	12.7%	Agorastos et al. (2015) [12]
Prevalence of HPV16 and/or 18	3.9%	Agorastos et al. (2015) [12]
Prevalence of CIN1	2.1%	Agorastos et al. (2015) [12]
Prevalence of CIN2	0.7%	Agorastos et al. (2015) [12]
Prevalence of CIN3	0.4%	Agorastos et al. (2015) [12]
Prevalence of invasive cervical cancer	0.053%	[13]

* All high risk genotypes except the 16 and 18

All costs were discounted at 3.5% and included all interventions reimbursed by the social insurance fund (EOPYY) for the diagnosis and the treatment of CIN and invasive cervical cancer. The HPV test is currently partially reimbursed by the social insurance fund, as users’ coinsurance rate has been set at 15% (12€). However, the analysis is conducted by estimating the economic impact in both the scenarios of partial and full reimbursement. On the contrary, cytology-based screening is fully reimbursed and is typically offered by the employment of two different examinations, i.e. 1) cytological examination of cervicovaginal smear, and 2) particular examination of endocervical smear, which are charged separately at 6.66 euros each (13.32€ in total). Unit costs for the resources used were obtained from the official price lists [39] (Table 3).

**Table 3:** Cost data.

Cost and resource utilization	Base case	Range	References
Screening			
Office visit (routine/repeat screening) (€)	10.00	8.00–12.00	EOPYY (2015)[39]
Office visit (diagnostic follow up) (€)	10.00	8.00–12.00	EOPYY (2015)[39]
Cytology test (liquid based) (€)	13.32	10.66–15.98	EOPYY (2015)[39]
Cytology test (conventional) (€)	13.32	10.66–15.98	EOPYY (2015)[39]
Cytology test additional cost of abnormal test (€)*	13.32	10.66–15.98	EOPYY (2015)[39]
HPV test(€)†	68.00	54.40–81.60	EOPYY (2015)[39]
HPV test(€)‡	80.00	64.00–96.00	EOPYY (2015)[39]
Linear array HPV genotyping test (€)†	68.00	54.40–81.60	EOPYY (2015)[39]
Linear array HPV genotyping test (€)‡	80.00	64.00–96.00	EOPYY (2015)[39]
Diagnosis			
Colposcopy plus biopsy (€)	38.74	30.99–46.49	EOPYY (2015)[39]
Treatment			
Treatment for CIN 2+ (€) **	1,533.02	1,226.41–1,839.62	Diaz et al. (2010) [40]
Treatment of invasive cervical cancer (€)**	20,572.60	16,458.08–24,687.12	Diaz et al. (2010) [40]

* The cytology test’s additional cost of an abnormal test equals the full cost of a conventional or liquid based cytology test

^†^The cost reimbursed by the social insurance fund excluding user’s copayment.

^‡^The cost if fully reimbursed by social insurance

**Cost were drawn from Diaz et al. (2010) and extrapolated to 2017 Greek values.

For strategies with the HPV test with simultaneous HPV 16 and 18 genotyping—no additional cost for reflex genotyping was included. In the absence of relevant data, the cost of treating cervical intraepithelial neoplasia (CIN2+) and cervical cancer was based on the respective Spanish costs [40], which were converted to 2017 Greek values using the relevant Consumer Price Index and Purchasing Power Parity exchange rate [41]. Both one-way sensitivity analysis (OWSA) and probabilistic sensitivity analysis (PSA), using Monte Carlo simulation (5,000 iterations), were performed for all model’s parameters. The clinical variables tested range was drawn from the HERMES study [12]; the cost, the epidemiology and natural history parameters range was ±20%, ±10% and ±5%, respectively (S1 Table). The adherence and compliance rate ranged from 20% to 80% (S1 Table). The distributions assumed for the above variables were lognormal for the clinical inputs, gamma for costs, normal for the epidemiology parameters and the compliance rate and beta for the natural history parameters.

### Ethics statement

The present study is an economic modelling study, which employs published data from the literature. In this context, it does not involve human participants and there were no direct or indirect interactions with patients or human specimens or tissue. The clinical data that were used (Table 1) were drawn from the study of Agorastos et al. [12], which has been reviewed and approved by an institutional review board (Ethical Committee of the Aristotle University of Thessaloniki). The respective Ethics Statement and the Ethical committee's Protocol number can be found in the published article [12].

## Results

The comparison of the under investigation strategies reveals that current practice (annual cytology) is inferior with regards to the incident of cancer and mortality due to missed disease from the majority of the comparators. Table 4 summarizes the results ranking all strategies from most to least effective according to their impact on cervical cancer incidence. When disease incidences are considered, five of the strategies appear to be less effective than current practice. Optimal effectiveness is demonstrated by the strategies that offer the HPV test triennially with genotyping either as co-testing with cytology or alone as a primary screening method. Compared to current practice, these strategies appear to decrease cervical cancer incidence by 32.2% (-7.5 incidence annually). This is in contrast to the strategies that include HPV testing–as co-testing or alone–without genotyping and the strategy with cytology alone every three years, which are estimated to provide worse outcomes, as the incidence of cancer appear to increase from 8.6% to 35.6% compared to annual cytology (Table 4).

**Table 4:** Annual cervical cancer mortality and incidence resulting from different screening strategies.

Screening strategies	Clinical impact					
	Cancers detected (%)	CIN 2+ detected (%)	Annual cervical cancer mortality*	Annual cervical cancer mortality compared to cytology alone (1 year)	Annual incidence of cervical cancer*	Annual incidence of cervical cancer compared to cytology (1 year)
**HPV test 16/18 genotyping (3 years)**	94.1%	88.7%	3.7	-73.2% (-10.1)	15.8	-32.2% (-7.5)
**HPV test reflex genotyping (3years)**	94.1%	88.7%	3.7	-73.2% (-10.1)	15.8	-32.2% (-7.5)
**Co-testing with cytology & HPV 16/18 genotyping (3 years)**	94.5%	89.6%	3.7	-73.2% (-10.1)	15.8	-32.2% (-7.5)
**Co-testing with reflex genotyping (3 years)**	94.5%	89.6%	3.7	-73.2% (-10.1)	15.8	-32.2% (-7.5)
**HPV test 16/18 genotyping (5 years)**	94.1%	88.7%	6.1	-55.8% (-7.7)	22	-5.6% (-1.3)
**HPV test reflex genotyping (5years)**	94.1%	88.7%	6.1	-55.8% (-7.7)	22	-5.6% (-1.3)
**Co-testing with cytology & HPV 16/18 genotyping (5 years)**	94.5%	89.6%	6.1	-55.8% (-7.7)	22	-5.6% (-1.3)
**Co-testing with reflex genotyping (5 years)**	94.5%	89.6%	6.1	-55.8% (-7.7)	22	-5.6% (-1.3)
**Cytology (1 year) (current practice)**	58.5%	56.4%	13.8	0.0	23.3	0.0
**Co-testing with cytology & HPV test (no genotyping) (3 years)**	74.6%	70.5%	10.7	- 22.5% (-3.1)	25.3	+8.6% (2.0)
**HPV test (no genotyping) (3 years)**	72.6%	67.9%	11.2	-18.8% (-2.6)	25.8	+10.7% (2.5)
**Co-testing with cytology & HPV test (no genotyping) (5 years)**	74.6%	70.5%	13.1	-5.1% (-0.7)	30.4	+30.5% (7.1)
**HPV test (no genotyping) (5 years)**	72.6%	68.0%	13.7	-0.7% (-0.1)	30.9	+32.6% (7.6)
**Cytology (3 years)**	58.5%	56.4%	15.9	+15.2% (2.1)	31.6	+35.6% (8.3)

*Impact of screening strategy and interval on missed disease and resulting progression.

With regards to cervical cancer deaths, it appears that all but three strategies have a significant positive impact on mortality from missed cases (Table 4). The most effective strategies decrease mortality by 73.2% and these are the triennial strategies, which include HPV testing with simultaneous or reflex genotyping. These are followed by the same strategies with 5 year screening interval which offer a mortality reduction of 55.8%.The less effective ones, are those offering cytology alone every 3 years (15.2% mortality increase) and those which include HPV testing with no genotyping, in a 5 year screening interval which reduce mortality by 0.7% and 5.1%. In more detail, cervical cancer deaths appear to decrease to 3.7 annually (10.1 deaths less than current practice) by the adoption of the aforementioned most effective strategies. Quinquennial co-testing with no genotyping and HPV testing alone with no genotyping appear to have a minor impact on mortality (-0.7 and -0.1 deaths compared to current practice) and triennial cytology is estimated to increase mortality by 2 deaths annually.

From a strict economic perspective, this analysis proves that, in most of the cases, current practice consumes more resources for inferior outcomes in both the partial and the full reimbursement scenarios, leading to inefficient resource allocation. Irrespective of the outcomes generated, under the partial reimbursement scenario all strategies appear to provide savings to social insurance, apart from those that are offered triennially and incorporate reflex genotyping. When full reimbursement is applied, the co-testing strategies with 16/18 genotyping and without genotyping provided every three years are added to the strategies that demand more resources than annual cytology.

**Fig 7:**
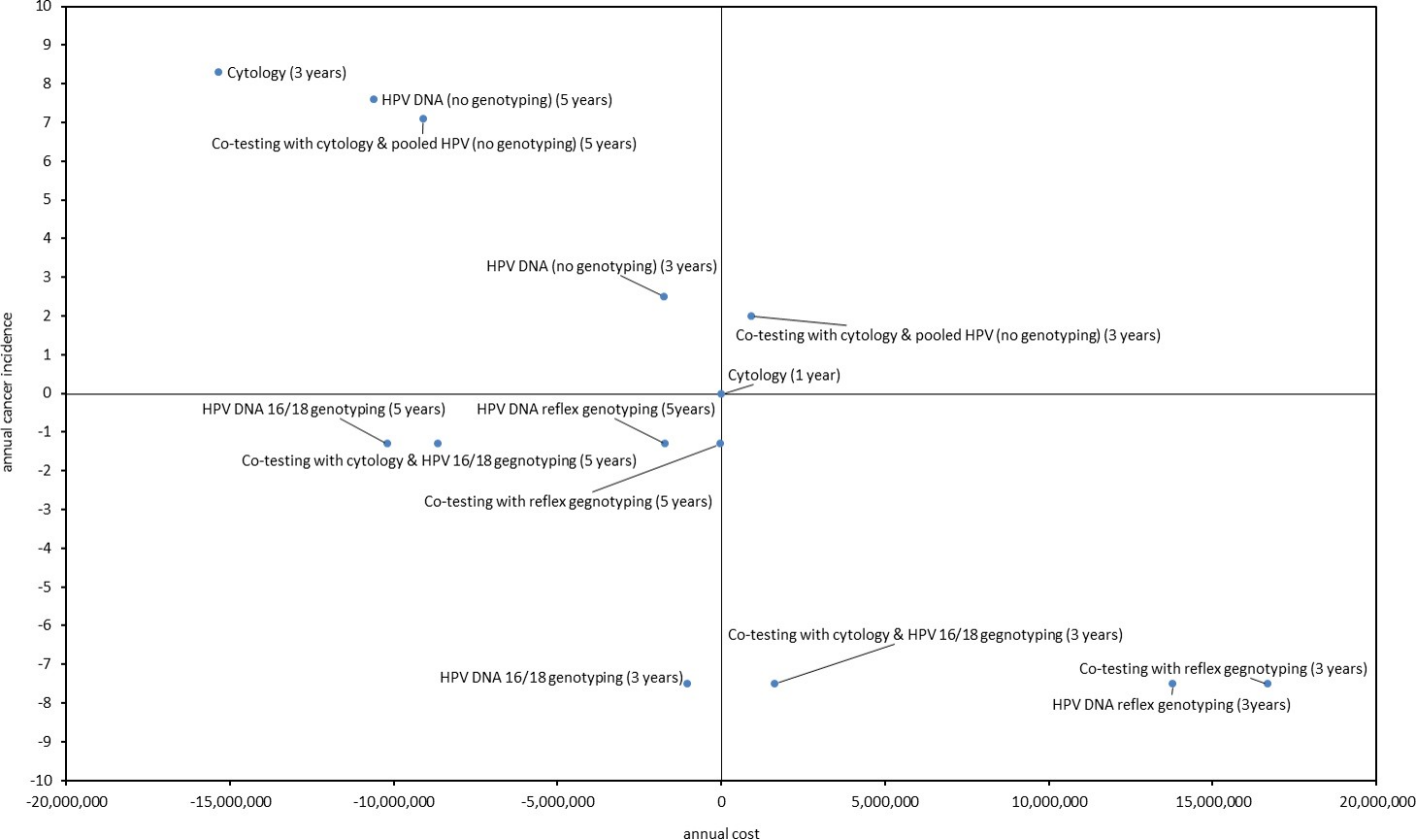
Annual cost and cancer incidence of all screening strategies compared to current practice.

**Fig 8:**
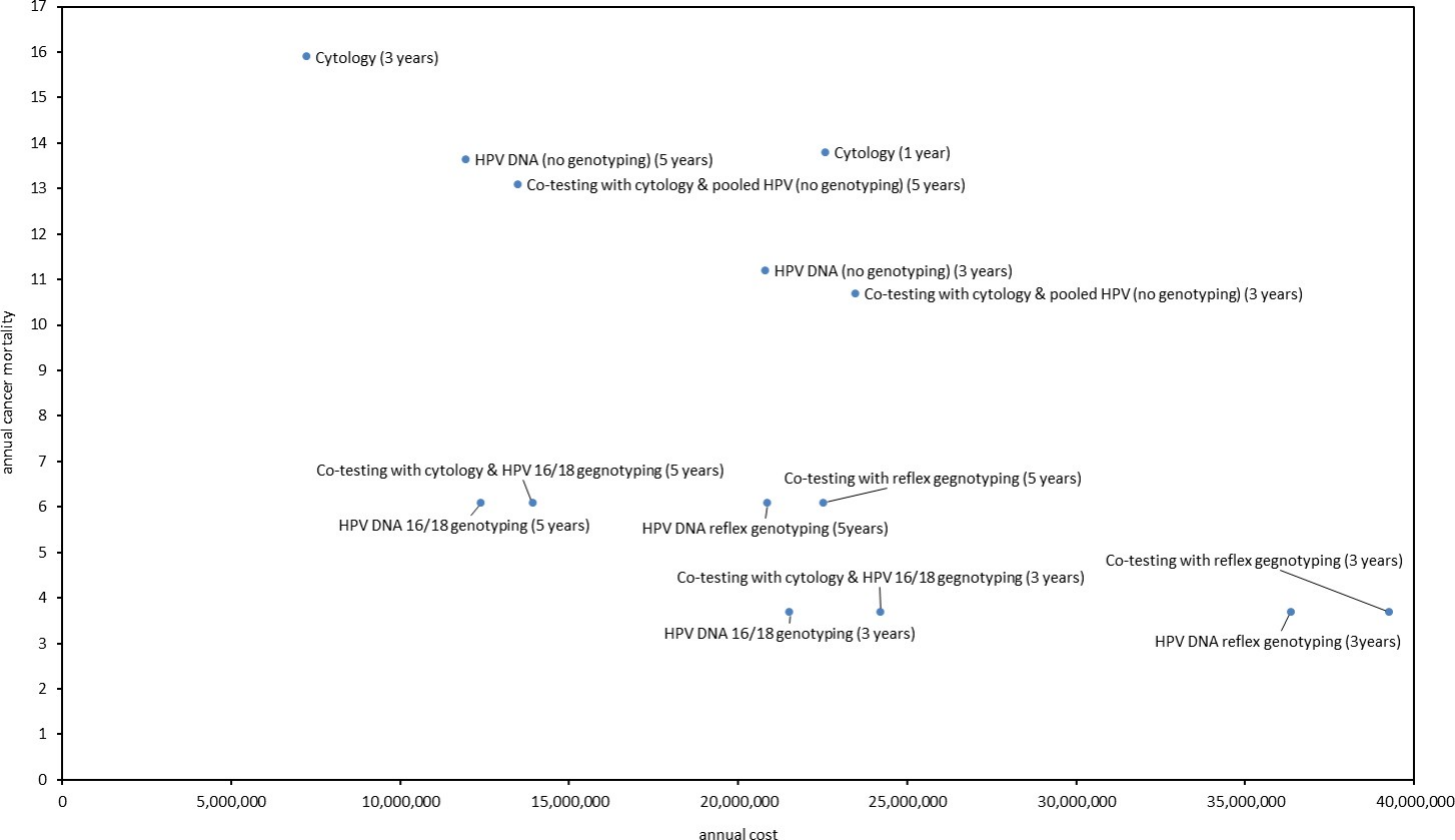
Annual cost and cancer mortality of all screening strategies.

By evaluating cost and outcomes it becomes evident that annual cytology is dominated by the majority of screening strategies. All strategies that include genotyping show better efficacy in reducing cancer incidence and among them, the optimum outcomes are generated by the screening strategies that are offered triennially and are accompanied by HPV genotyping. However, even in the scenario of full HPV test reimbursement, the strategy of HPV test with simultaneous 16/18 genotyping every 3 years appears to be a cost saving strategy (-1,050,188 euros) that provides maximum cancer protection by considering both annual cancer incidence and mortality (Tables 4 and 5 and Figs 7 and 8). Adding cytology to the HPV test at primary screening, and/or substituting simultaneous 16/18 genotyping with reflex genotyping, increases the annual cost of the program from 19.2 million euros to 34.7 million euros without evidence of outcomes improvement. On the other hand, the extension of the screening interval to 5 years to the above strategies increases savings–apart from co-testing with reflex genotyping—for the social insurance budget, though these come by forgoing cervical cancer outcomes. Among them, the strategy of HPV test with simultaneous 16/18 genotyping every 5 years provides the maximum annual savings (10,187,916 euros) without trading off clinical outcomes. When cost per death averted and cost per incidence reduced are considered, the above strategy appears to be more attractive compared to triennial HPV with simultaneous 16/18 genotyping as the respective ratios reveal that the former saves 1,323,106€/death averted and 7,836,858 €/incidence reduced when the latter saves 103,979€/death averted and 140,025€/incidence reduced compared to current practice (Table 6).

**Table 5:** Annual cost of screening strategies under partial and full HPV test reimbursement.

Screening strategies	Economic impact									
** **	Partial reimbursement (85%)					Full reimbursement (100%)				
** **	Annual total cost	Annual screening cost	Annual diagnostic cost	Annual treatment cost	Annual cost comapred with cytology (1 year)	Annual total cost	Annual screening cost	Annual diagnostic cost	Annual treatment cost	Annual cost comapred with cytology (1 year)
**HPV test 16/18 genotyping (3 years)**	19,241,165	15,017,256	357,422	3,866,487	-3,325,970	21,516,948	17,293,038	357,422	3,866,487	- 1,050,188
**HPV test reflex genotyping (3years)**	31,842,217	27,618,308	357,422	3,866,487	9,275,082	36,341,714	32,117,805	357,422	3,866,487	13,774,579
**Co-testing with cytology & HPV 16/18 gegnotyping (3 years)**	21,938,911	17,371,974	677,014	3,889,923	-628,224	24,197,170	19,630,233	677,014	3,889,923	1,630,035
**Co-testing with reflex genotyping (3 years)**	34,735,713	30,168,776	677,014	3,889,923	12,168,578	39,252,231	34,685,294	677,014	3,889,923	16,685,096
**HPV test 16/18 genotyping (5 years)**	11,077,501	8,590,137	205,304	2,282,059	-11,489,634	12,379,219	9,891,856	205,304	2,282,059	- 10,187,916
**HPV test reflex genotyping (5years)**	18,284,713	15,797,350	205,304	2,282,059	-4,282,422	20,858,292	18,370,929	205,304	2,282,059	- 1,708,843
**Co-testing with cytology & HPV 16/18 genotyping (5 years)**	12,621,056	9,937,151	387,209	2,296,696	-9,946,079	13,912,814	11,228,909	387,209	2,296,696	- 8,654,321
**Co-testing with reflex genotyping (5 years)**	19,941,019	17,257,114	387,209	2,296,696	-2,626,116	22,524,536	19,840,631	387,209	2,296,696	- 42,599
**Cytology (1 year)**	22,567,135	14,443,447	665,645	7,458,043	0	22,567,135	14,443,447	665,645	7,458,043	0
**Co-testing with cytology & HPV test (no genotyping) (3 years)**	21,192,982	17,526,116	455,906	3,210,961	-1,374,153	23,470,953	19,804,087	455,906	3,210,961	903,817
**HPV test (no genotyping) (3 years)**	18,515,026	15,231,454	152,447	3,131,124	-4,052,109	20,809,265	17,525,693	152,447	3,131,124	- 1,757,870
**Co-testing with cytology & HPV test (no genotyping) (5 years)**	12,166,836	10,024,454	261,001	1,881,381	-10,400,299	13,469,773	11,327,391	261,001	1,881,381	-9,097,362
**HPV test (no genotyping) (5 years)**	10,633,042	8,712,125	87,938	1,832,979	-11,934,093	11,945,232	10,024,315	87,938	1,832,979	-10,621,903
**Cytology (3 years)**	7,224,599	4,583,723	211,275	2,429,601	-15,342,537	7,224,599	4,583,723	211,275	2,429,601	-15,342,537

**Table 6:** Incremental cost per outcome under partial and full HPV test reimbursement.

Screening strategies	Cost per outcome															
	Partial reimbursement (85%)								Full reimbursement (100%)							
	Total cost	Deaths averted	Incidence reduced	Δ Cost	Δ Deaths	Δ incidence	Cost / Death averted	Cost/ Incidence reduced	Total cost	Deaths averted	Incidence reduced	Δ Cost	Δ Deaths	Δ incidence	Cost / Death averted	Cost/ Incidence reduced
**Cytology (1 year)**	**22,567,135**	13.8	23.3	-	**- **	**- **	** -**	**- **	22,567,135	13.8	23.3	-	-	** -**	** -**	**- **
**HPV test 16/18 genotyping (3 years)**	**19,241,165**	3.7	15.8	-3,325,970	-10.1	-7.5	329,304	443,463	21,516,948	3.7	15.8	-1,050,187	-10.1	-7.5	103,979	140,025
**HPV test reflex genotyping (3years)**	31,842,217	3.7	15.8	9,275,082	-10.1	-7.5	-918,325	-1,236,678	36,341,714	3.7	15.8	13,774,579	-10.1	-7.5	-1,363,820	-1,836,611
**Co-testing with cytology & HPV 16/18 gegnotyping (3 years)**	21,938,911	3.7	15.8	-628,224	-10.1	-7.5	62,200	83,763	24,197,170	3.7	15.8	1,630,035	-10.1	-7.5	-161,390	-217,338
**Co-testing with reflex genotyping (3 years)**	34,735,713	3.7	15.8	12,168,578	-10.1	-7.5	-1,204,810	-1,622,477	39,252,231	3.7	15.8	16,685,096	-10.1	-7.5	-1,651,990	-2,224,679
**HPV test 16/18 genotyping (5 years)**	11,077,501	6.1	22	-11,489,634	-7.7	-1.3	1,492,160	8,838,180	12,379,219	6.1	22	-10,187,916	-7.7	-1.3	1,323,106	7,836,858
**HPV test reflex genotyping (5years)**	18,284,713	6.1	22	-4,282,422	-7.7	-1.3	556,159	3,294,171	20,858,292	6.1	22	-1,708,843	-7.7	-1.3	221,928	1,314,495
**Co-testing with cytology & HPV 16/18 genotyping (5 years)**	12,621,056	6.1	22	-9,946,079	-7.7	-1.3	1,291,699	7,650,830	13,912,814	6.1	22	-8,654,321	-7.7	-1.3	1,123,938	6,657,170
**Co-testing with reflex genotyping (5 years)**	19,941,019	6.1	22	-2,626,116	-7.7	-1.3	341,054	2,020,089	22,524,536	6.1	22	-42,599	-7.7	-1.3	5,532	32,768
**Co-testing with cytology & HPV test (no genotyping) (3 years)**	21,192,982	10.7	25.3	-1,374,153	-3.1	2.0	443,275	-687,077	23,470,953	10.7	25.3	903,818	-3.1	2.0	-291,554	451,909
**HPV test (no genotyping) (3 years)**	18,515,026	11.2	25.8	-4,052,109	-2.6	2.5	1,558,503	-1,620,844	20,809,265	11.2	25.8	-1,757,870	-2.6	2.5	676,104	-703,148
**Co-testing with cytology & HPV test (no genotyping) (5 years)**	12,166,836	13.1	30.4	-10,400,299	-0.7	7.1	14,857,570	-1,464,831	13,469,773	13.1	30.4	-9,097,362	-0.7	7.1	12,996,231	-1,281,319
**HPV test (no genotyping) (5 years)**	10,633,042	13.7	30.9	-11,934,093	-0.1	7.6	119,340,930	-1,570,275	11,945,232	13.7	30.9	-10,621,903	-0.1	7.6	106,219,030	-1,397,619
**Cytology (3 years)**	7,224,599	15.9	31.6	-15,342,536	2.1	8.3	-7,305,970	-1,848,498	7,224,599	15.9	31.6	-15,342,536	2.1	8.3	-7,305,970	-1,848,498

The difference in the total annual cost between the strategies is attributed to the compared screening algorithms’ clinical effectiveness and cost. All HPV strategies detect more CIN2+ (Table 4) and thus provide the health system with the opportunity to treat these cases earlier and subsequently to increase cost as more cases are being detected and have to be managed. In parallel, the social insurance fund avoids later costs that are associated with CIN2+ cases progression to CC, which is depicted by the decrease of the annual cancer mortality due to missed disease that the HPV strategies achieve. The above is shown in Table 5 where the majority of the strategies appear to incur higher annual and diagnostic costs, but lower treatment costs than current practice. In addition, adding cytology to the HPV test in first line screening and using reflex genotyping, instead of simultaneous 16/18 genotyping, adds substantial cost to the system without impacting the outcomes of the respective screening algorithm.

**Fig 9:**
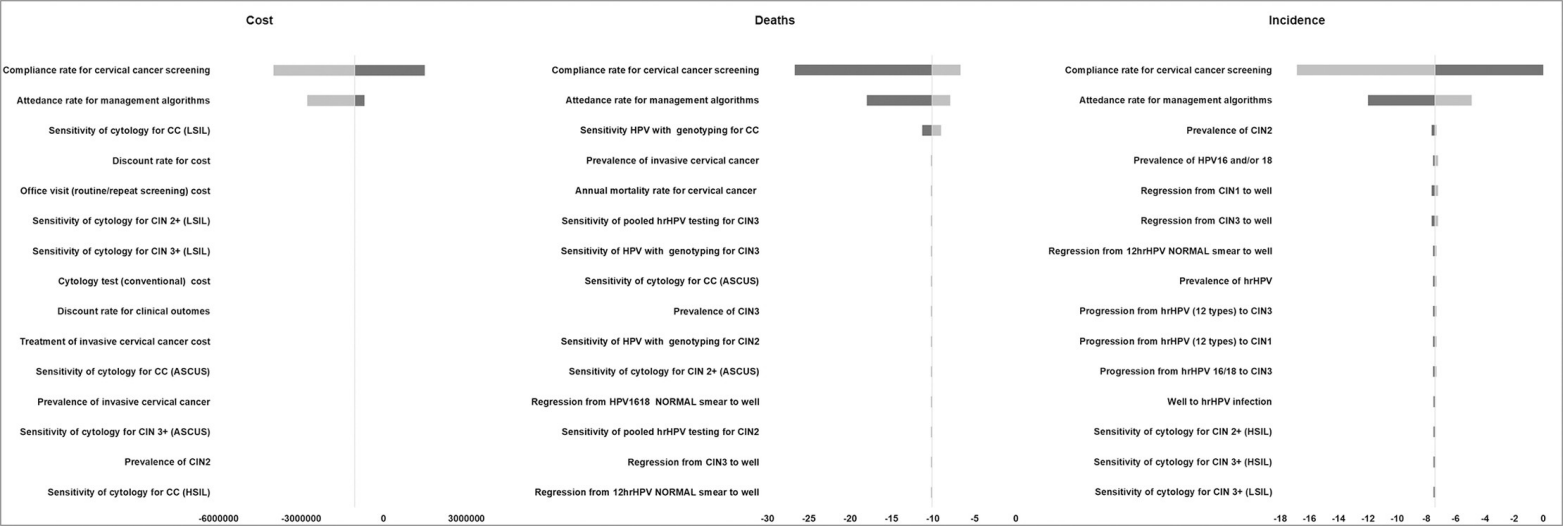
One way sensitivity analysis results of 3-year HPV testing with simultaneous 16/18 genotyping versus annual cytology.

**Fig 10:**
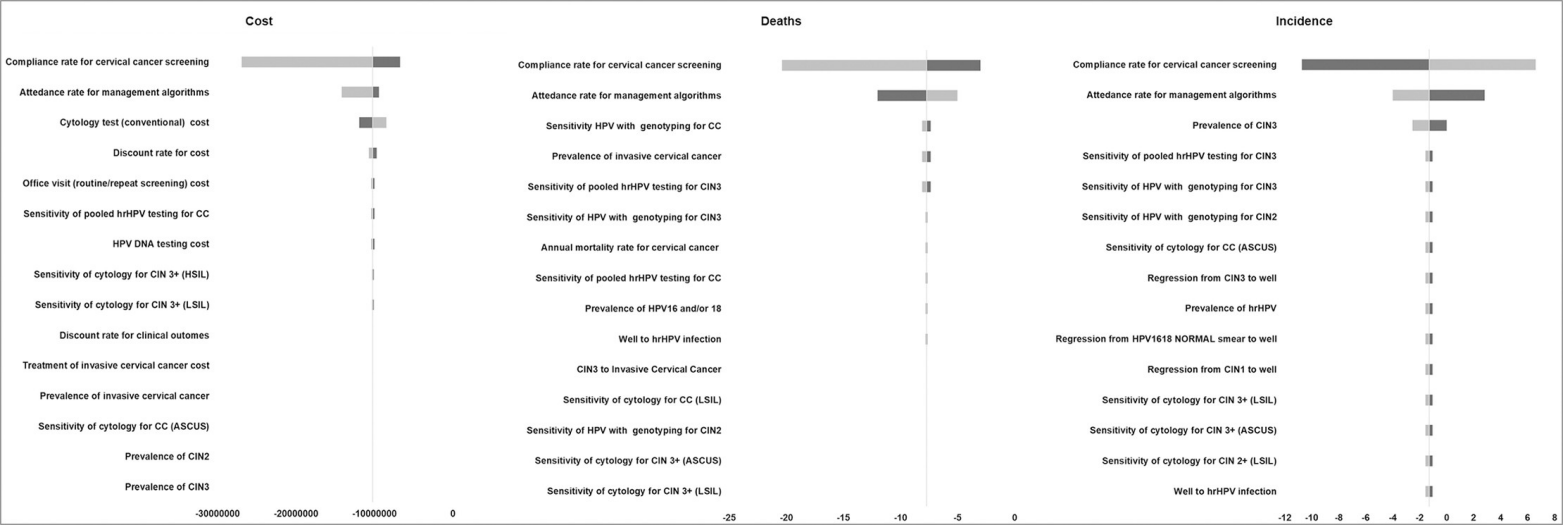
One way sensitivity analysis results of 5-year HPV testing with simultaneous 16/18 genotyping versus annual cytology.

**Fig 11:**
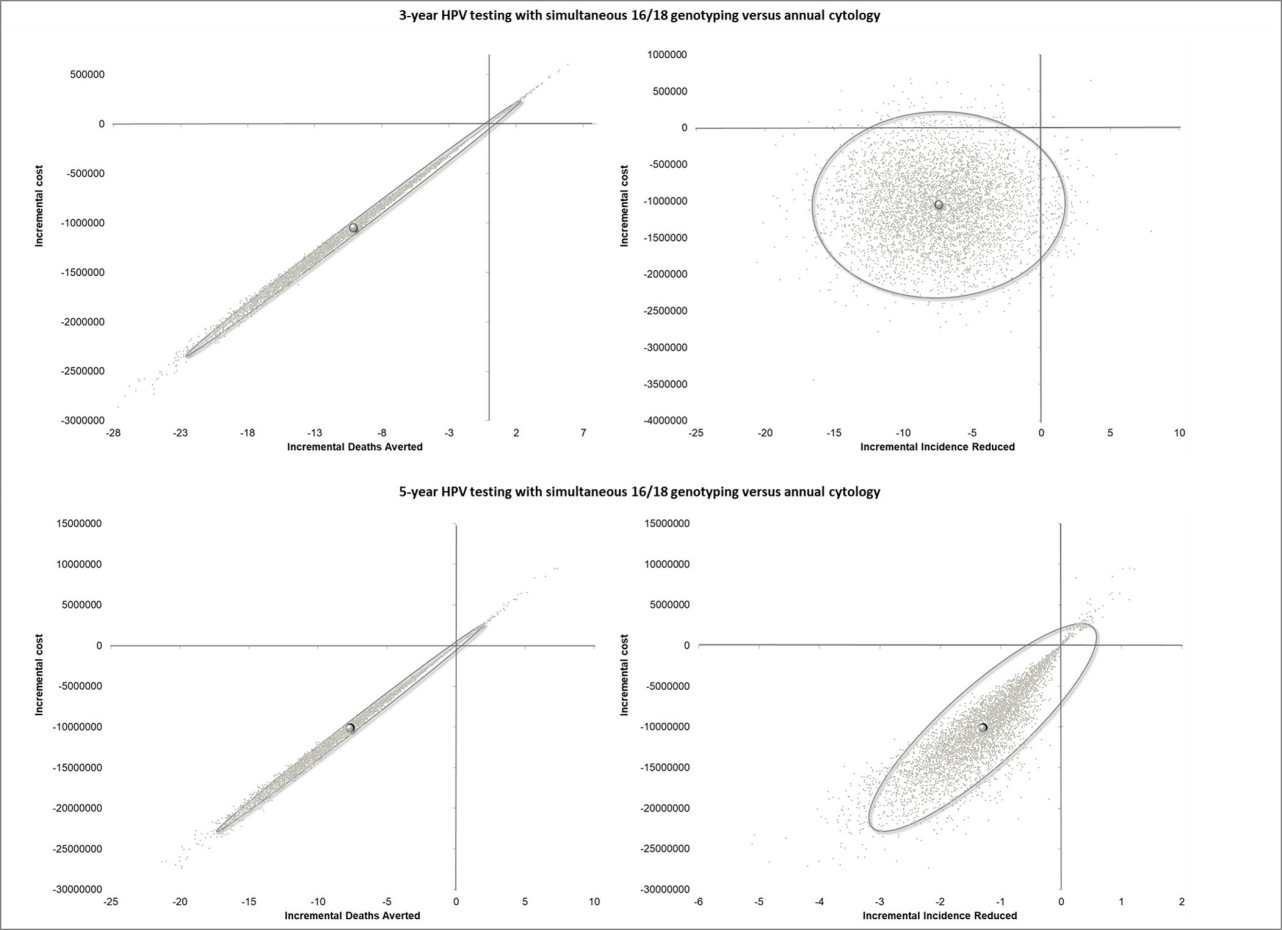
Probablisitic Sensitivity Analysis results of 3-year and 5-year HPV testing with simultaneous 16/18 genotyping versus annual cytology.

Both OWSA and PSA were conducted for all screening strategies and proved that the results of the model are robust. The outcomes of both the sensitivity analyses for the screening strategies of 3-year and 5-year HPV testing with simultaneous 16/18 genotyping versus annual cytology are presented in Figs 9, 10 and 11 and in S1 and S2 Figs. As expected, the OWSA shows that the incremental cost, the deaths averted and the incidence reduced of the respective strategies are sensitive mostly to the compliance rate and the attendance rate for management algorithms. In addition, the 3-year strategy’s incremental cost appears to be sensitive to cycology’s sensitivity for invasive cervical cancer (ICC) and the deaths averted to the sensitivity of the HPV test with genotyping for ICC (Fig 9). On the other hand, the 5-year strategy’s incremental cost appears to be sensitive to the cytology’s cost while the deaths averted and the incidence reduced to the sensitivity of the HPV test with genotyping for ICC and the prevalence of CIN3, respectively (Fig 10). However, the PSA results reveal that the results of the analysis regarding the aforementioned strategies are robust in 95% confidence interval (Fig 11).

## Discussion

The analysis supports that from a public health perspective, adopting the HPV test with genotyping (simultaneous or reflex) with or without cytology in first line screening for cervical cancer is more clinically effective than cytology alone. However, from the most effective strategies, the screening algorithm of triennial HPV testing with simultaneous 16/18 genotyping appear to provide to the social insurance fund the opportunity to save resources (-1,050,188€) by achieving the optimum outcomes, at the same time. These savings are realized in both the partial and full reimbursement of the HPV test scenarios, though higher in the former (-3,325,970€).

Nevertheless, it appears that the aforementioned strategy when offered quinquennially instead of triennially still provide better outcomes than current practice, but it generates significant higher savings (up to 10 million euros) at the expense of lower outcomes than when offered more frequently. The dilemma the decision makers face in such cases is whether the increased savings can justify or compensate for the lower health outcomes they come with. From an economist’s point of view, this decision could be justified in the case that these savings can be invested in another intervention that will provide more health outcomes than those lost from this choice. An example of this could probably be investing in interventions to increase participation in the HPV vaccination program. However, this still remains a political decision and is outside of the scope of this study. Despite that, the adoption of 5 year screening interval is supported by relevant studies that present efficiency gains and outcomes improvement [42, 43].

In contrast to cost-utility analyses, budget impact and cost per death averted or per incidence reduced analyses are not usually accompanied by an established national threshold regarding the additional cost for the outcomes provided and thus, efficiency in such cases is mostly subject to current political views and the economic environment. Though, from an economic perspective, the current analysis provides strong arguments towards the adoption of HPV test with simultaneous 16/18 genotyping every 3 or 5 years and is in parallel supported by increasing evidence that adopting the HPV test as a primary screening method is a cost-effective or even cost-saving option compared to cytology [44–51]. Moreover, given that the 4 different types of HPV strategies–HPV with or without cytology in first line screening and with simultaneous or reflex genotyping—are equally effective, when identical screening intervals are applied, this analysis could also serve as a cost-minimization analysis between HPV test 16/18 genotyping, HPV test reflex genotyping, co-testing with cytology & HPV 16/18 genotyping and co-testing with reflex genotyping. In this context, the former clearly dominates the others, as it results in the same outcomes with substantially fewer resources.

By considering Cochrane’s and Holland’s screening program criteria [17, 18], the HPV test seems to be superior or at least equal to cytology in most of the evaluation criteria. In contrast to cytology, the HPV test is simpler to perform and interpret due to the fact that the sample analysis is an automated method, it does not require significant expertise and thus, the results are not subjective as in the case of cytology [52].

Regarding its acceptability by the individuals, the HPV test can be safely assumed to be as acceptable as the cytology, due to the fact that women are going through the same procedure in order to perform the test.

In terms of test’s accuracy, even though the HPV test and cytology examine different clinical outcomes, the HPV test initiated screening algorithms proved to be more accurate than cytology alone. This is attributed to HPV test’s superior performance and to the fact that in contrast to cytology, it provides the opportunity to better identify individuals’ risk of developing CIN3 [13–16, 53] in the upcoming 5 to 15 years and hence, optimizes the cancer risk management.

Repeatability is an additional criterion under which the tests should be evaluated and as described above the HPV test is more capable of providing consistent results in repeated trials compared to cytology for which interpretation involves a significant level of subjectivity as shown in relevant studies [52, 54].

In terms of the under comparison tests’ performance, it has been demonstrated in numerous clinical studies that HPV test dominates cytology when sensitivity is examined, but shows slightly lower specificity [10–16]. However, HPV test’s specificity appears not to impact the screening algorithm’s budget impact as shown by the OWSA conducted (S1 Fig, S2 Fig).

Based on the discussion above, it becomes evident that HPV 16/18 genotyping according to Cochrane’s and Holland’s criteria is superior to cytology. On the contrary, any discussion regarding the adoption of an HPV test with reflex genotyping or co-testing with simultaneous or reflex genotyping appears to be redundant, since these strategies demand more resources compared to current practice for the same outcomes that the HPV 16/18 genotype provides.

Adjusting the discussion to the Greek public health setting, the adoption of HPV 16/18 genotyping provides the opportunity to improve cervical cancer prevention even with low and inadequate levels of screening compliance rates, as those currently observed. Individuals’ adherence to all screening guidelines in Greece is quite low over time and hence, from a health policy perspective several interventions should be adopted in order to optimize secondary prevention. In line with the above, any intervention that improves access, awareness or screening effectiveness even for the few screened, should be placed as a priority in the health policy agenda. This is also enforced by the fact that in times of economic turmoil and fiscal restrictions health care resources should be shifted to primary care and public health in order to increase the efficiency of the resources invested in health care and to optimize population health status [55–57]. In addition, Greece appears to be missing the opportunity of primary prevention of cervical cancer. The vaccine is offered free of charge to girls 11–18 years old and to three special groups of 18–26 years of age that are HIV positive, immunocompromised or are men having sex with men. Despite that, the vaccination coverage is far below the optimal (27%) [58]. The latter also stress the need of a more efficient secondary prevention strategy in order to control the burden of cervical cancer which could partly be offered by a more effective screening test like the case of HPV 16/18 genotyping.

HPV vaccination can contribute to the reduction of HPV infection and consequently to the burden of CIN2+ and CC. Therefore, as vaccination coverage increases, the marginal benefit of screening decreases due to the fact that fewer women will be tested positive to the virus. In this sense, this could be perceived as limitation of the present study as the vaccine’s impact has not been taken into account. Though, the vaccination coverage in Greece is quite low and thus the value of screening remains high. Nonetheless, regardless of the vaccination coverage, HPV screening still appears to be of great importance and of good value for money versus cytology [59]. However, as vaccination increases, screening frequency as long as screening age-group should be revaluated. In this context, the strategy of HPV test with simultaneous 16/18 genotyping every 5 years will become even more attractive.

In the absence of relevant data, the screening compliance rate and the attendance rate at re-testing have been assumed equal. Given that in light of positive or equivocal results, the attendance rate at retesting can be assumed increased, this could be seen as a limitation and a parameter that impacts the results of the model, as well, as shown by the OWSA. However, the PSA conducted included both high (80%) and low (20%) attendance rates and revealed that the model’s results remain robust.

The prevalence of CC was assumed equal to that observed in the ATHENA trial [13] as in the local HERMES [12] trial no CC cases were detected. In addition, due to the absence of data regarding the management and treatment cost of CINs and CC the relevant cost inputs were inferred by adjusting respective data from Spain, as described in the methods section, which is a practice that is being employed in similar cases [46]. However, all the above parameters have been included in both the OWSA and the PSA and show that do not impact the results of the model.

In addition, the present study estimates a specific incidence outcome which is (the difference in) incidence and mortality due to missed disease and resulting progression from the different screening strategies under investigation. Currently, there are no relevant local real world data of such an estimate (i.e. incidence and mortality due to missed disease) that could be used to compare our results and to validate the disease natural history model in this way.

The transition probabilities used for CIN are not stratified by HPV type due to our currently limited relevant knowledge and as Wright et al.[13] argue in a relevant analysis, the effectiveness of HPV 16/18 genotyping in this case could be underestimated. This assumption could also serve as a limitation of the present study however; it appears that probably leads to an overestimation of the budget impact of the HPV genotyping strategy.

The present study was conducted for both HPV partial reimbursement (current reimbursement status) and full reimbursement scenarios. In this context, the adherence rate in HPV screening under the scenario of partial reimbursement may be overestimated as it has been proved that co-payment negatively impacts health services utilization [60–62]. For this reason, the analysis was focused on the full reimbursement scenario for eliminating such concerns and more importantly to highlight the importance of full reimbursement of screening programs in order to minimize barriers in access and thus maximize adherence and outcomes.

Finally, despite the fact that HPV screening is being discussed for age groups >25 and >30 years old, the present study examined the first scenario as there is evidence to suggest that HPV screening is more effective when it begins earlier, at the age of 25 [13], which is also supported by experts views [63] and due to the fact that the HERMES study [12], from which the clinical data were drawn, was also carried out for women >25 years of age.

Screening with the HPV test is an issue that has received a lot of international attention from academic societies, physicians and health care systems. In this direction, the international discussion of whether the HPV test should replace cytology as a primary screening method for cervical cancer concludes in favor of the former [64–68]. In line with the above, numerous countries of different economic status including the Netherlands [69], Australia [70], New Zealand [71], Turkey [72], Argentina, Mexico [73] and Italy [74], have decided to adopt the test as a first line screening method.

Our analysis appears to support the outcome of the international dialogue and sets ground to the point that HPV screening is the most effective intervention to adopt in a National Screening Program. Primary screening with HPV test 16/18 genotyping alone every 3 or 5 years is the strategy that provides the opportunity of optimal resource allocation and health outcomes improvement. However, the adoption of the most cost-effective strategy is a health policy decision, which will be based on the current economic environment and the cost and benefits trade off. In this context, the present study could serve as an input to the decision making process and contribute to the discussion regarding cervical cancer prevention in Greece.

## Supporting information

S1 Table All model parameters and sensitivity analyses range. (DOCX)

S1 Fig One-way sensitivity analysis results of 3-year HPV testing with simultaneous 16/18 genotyping versus annual cytology (all parameters results ranked according to their impact). (DOCX)

S2 Fig One-way sensitivity analysis results of 5-year HPV testing with simultaneous 16/18 genotyping versus annual cytology (all parameters results ranked according to their impact). (DOCX)

## References

[1] International Agency for Research on Cancer. 2018; Available from: http://globocan.iarc.fr.

[2] Organization for Economic Co-operation and Development. OECD statistics. 2015; Available from: http://stats.oecd.org/.

[3] World Health Organization. Global burden of disease. 2016; Available from: https://www.who.int/healthinfo/global_burden_disease/estimates/en/index1.html

[4] Papanicolaou GN. Atlas of Exfoliative Cytology. Boston, Massachusetts: Commonweath Fund University Press; 1954.

[5] Gakidou E, Nordhagen S, Obermeyer Z. Coverage of Cervical Cancer Screening in 57 Countries: Low Average Levels and Large Inequalities. PLoS Med.2008; 5(6): e132 10.1371/journal.pmed.0050132 18563963 PMC2429949

[6] Agorastos T, Chatzistamatiou K, Zafrakas M, Siamanta V, Katsamagkas T, Constantinidis TC, et al Epidemiology of HPV infection and current status of cervical cancer prevention in Greece: final results of the LYSISTRATA cross-sectional study. Eur J Cancer Prev. 2014;23:425–31. 10.1097/CEJ.0000000000000060 24977385

[7] zur Hausen H. Human papillomaviruses and their possible role in squamous cell carcinomas. Curr Top Microbiol Immunol. 1977;78:1–30. 10.1007/978-3-642-66800-5_1 202434

[8] Walboomers JM, Jacobs MV, Manos MM, Bosch FX, Kummer JA, Shah KV, et al Human papillomavirus is a necessary cause of invasive cervical cancer worldwide. J Pathol. 1999;189:12–9. 10.1002/(SICI)1096-9896(199909)189:1<12::AID-PATH431>3.0.CO;2-F 10451482

[9] De Vuyst H, Clifford G, Li N, Franceschi S. HPV infection in Europe. Eur J Cancer. 2009;45:2632–9. 10.1016/j.ejca.2009.07.019 19709878

[10] Whitlock EP, Vesco K.K., Eder M, JS, Senger CA, Burda BU. Liquid-based cytology and human papillomavirus testing to screen for cervical cancer: a systematic review for the U.S. Preventive Services Task Force. Ann Intern Med. 2011;155:687–97. 10.7326/0003-4819-155-10-201111150-00376 22006930

[11] Cuzick J, Szarewski A, Mesher D, Cadman L, Austin J, Perryman K, et al Long-term follow-up of cervical abnormalities among women screened by HPV testing and cytology—Results from the Hammersmith study. Int. J. Cancer. 2008;122:2294–300. 10.1002/ijc.23339 18240149

[12] Agorastos T, Chatzistamatiou K, Katsamagkas T, Koliopoulos G, Daponte A, Constantinidis T et al Primary screening for cervical cancer based on High-Risk Human Papillomavirus (HPV) detection and HPV16 and HPV18 genotyping, in comparison to cytology. PLoS One. 2015; 20;10(3):e0119755 10.1371/journal.pone.0119755 25793281 PMC4368762

[13] Wright TC, Stoler MH, Behrens CM, Sharma A, Zhang G, Wright TL. Primary cervical cancer screening with human papillomavirus: end of study results from the ATHENA study using HPV as the first-line screening test. Gynecol Oncol. 2015;136:189–97. 10.1016/j.ygyno.2014.11.076 25579108

[14] Gage JC, Katki HA, Schiffman M, Fetterman B, Poitras NE, Lorey T, et al Age-stratified 5-year risks of cervical precancer among women with enrollment and newly detected HPV infection. Int J Cancer. 2015;136:1665–71. 10.1002/ijc.29143 25136967 PMC4314342

[15] Ronco G, Dillner J, Elfström KM, Tunesi S, Snijders PJ, Arbyn M, et al Efficacy of HPV-based screening for prevention of invasive cervical cancer: follow-up of four European randomised controlled trials. Lancet. 2014; 383:524–32. 10.1016/S0140-6736(13)62218-7 24192252

[16] Elfström KM, Smelov V, Johansson AL, Eklund C, Nauclér P, Arnheim-Dahlström L, et al Long term duration of protective effect for HPV negative women: follow-up of primary HPV screening randomised controlled trial. BMJ. 2014;348: 10.1136/bmj.g130 24435414 PMC3898575

[17] Cochrane AL, Holland WW. Validation of screening procedures. British Medical Bulletin. 1971;27:3–8. 10.1093/oxfordjournals.bmb.a070810 5100948

[18] Holland WW, Stewart S, Masseria C. Policy brief: screening in Europe. Copenhagen: European Observatory on Health Systems and Policies; 2006.

[19] Hellenic Statistical Authority. 2016; Available from http://www.statistics.gr/statistics/pop.

[20] Kulasingam SL, Havrilesky LJ, Ghebre R, Myers ER. Screening for cervical cancer: a modeling study for the US Preventive Services Task Force. J Low Genit Tract Dis. 2013;17:193–202. 10.1097/LGT.0b013e3182616241 23519288 PMC3608928

[21] Kjær SK, Frederiksen K, Munk C, Iftner T. Long-term absolute risk of cervical intraepithelial neoplasia grade 3 or worse following human papillomavirus infection: role of persistence. J Natl Cancer Inst. 2010;102:1478–88. 10.1093/jnci/djq356 20841605 PMC2950170

[22] Khan MJ, Castle PE, Lorincz AT, Wacholder S, Sherman M, Scott DR, et al The elevated 10-year risk of cervical precancer and cancer in women with human papillomavirus (HPV) type 16 or 18 and the possible utility of type-specific HPV testing in clinical practice. J Natl Cancer Inst. 2005;97:1072–9. 10.1093/jnci/dji187 16030305

[23] Insinga RP, Dasbach EJ, Elbasha EH, Liaw KL, Barr E. Progression and regression of incident cervical HPV 6, 11, 16 and 18 infections in young women. Infect Agent Cancer. 2007; 10.1186/1750-9378-2-15 17626624 PMC2034372

[24] Insinga RP, Perez G, Wheeler CM, Koutsky LA, Garland SM, Leodolter S, et al Investigators. Incident cervical HPV infections in young women: transition probabilities for CIN and infection clearance. Cancer Epidemiol Biomarkers Prev. 2011;20:287–96. 10.1158/1055-9965.EPI-10-0791 21300618

[25] Kataja V, Syrjänen K, Mäntyjärvi R, Väyrynen M, Syrjänen S, Saarikoski S et al Prospective follow-up of cervical HPV infections: life table analysis of histopathological, cytological and colposcopic data. Eur J Epidemiol. 1989;51:1–7. 10.1007/BF00145037 2540024

[26] Holowaty P, Miller AB, Rohan T, To T. Natural history of dysplasia of the uterine cervix. J Natl Cancer Inst. 1999;91:252–58. 10.1093/jnci/91.3.252 10037103

[27] Matsumoto K, Yasugi T, Oki A, Fujii T, Nagata C, Sekiya S et al IgG antibodies to HPV16, 52, 58 and 6 L1-capsids and spontaneous regression of cervical intraepithelial neoplasia. Cancer Lett. 2006;231:309–13 10.1016/j.canlet.2005.02.023 16399232

[28] Guedes AC, Zeferino LC, Syrjänen KJ, Brenna SM. Short-term outcome of cervical intraepithelial neoplasia grade 2: considerations for management strategies and reproducibility of diagnosis. Anticancer Res. 2010;306:2319–23. 20651386

[29] Omori M, Hashi A, Nakazawa K, Yuminamochi T, Yamane T, Hirata S et al Estimation of prognoses for cervical intraepithelial neoplasia 2 by p16INK4a immunoexpression and high-risk HPV in situ hybridization signal types. Am J Clin Pathol. 2007;128:208–17. 10.1309/0UP5PJK9RYF7BPHM 17638654

[30] McCredie MR, Sharples KJ, Paul C, Baranyai J, Medley G, Jones RW et al Natural history of cervical neoplasia and risk of invasive cancer in women with cervical intraepithelial neoplasia 3: a retrospective cohort study. Lancet Oncol. 2008;9:425–34. 10.1016/S1470-2045(08)70103-7 18407790

[31] Sasieni P, Castanon A, Parkin DM. How many cervical cancers are prevented by treatment of screen-detected disease in young women? Int J Cancer. 2009;124:461–4. 10.1002/ijc.23922 18844220

[32] Goldie SJ, Kim JJ, Wright TC. Cost-effectiveness of human papillomavirus DNA testing for cervical cancer screening in women aged 30 years or more. Obstet Gynecol. 2004;103:619–31. 10.1097/01.AOG.0000120143.50098.c7 15051550

[33] Mandelblatt JS, Lawrence WF, Womack SM, Jacobson D, Yi B, Hwang YT et al Benefits and costs of using HPV testing to screen for cervical cancer. JAMA. 2002;287:2372–81. 10.1001/jama.287.18.2372 11988058

[34] Insinga RP, Dasbach EJ, Elbasha EH. Epidemiologic natural history and clinical management of Human Papillomavirus (HPV) Disease: a critical and systematic review of the literature in the development of an HPV dynamic transmission model. BMC Infect Dis. 2009; 10.1186/1471-2334-9-119 19640281 PMC2728100

[35] National Cancer Institute. 2016; https://seer.cancer.gov/data/access.html.

[36] Bulkmans NW, Berkhof J, Bulk S, Bleeker MC, van Kemenade FJ, Rozendaal L et al High-risk HPV type-specific clearance rates in cervical screening. Brit J Cancer. 2007;96:1419–24. 10.1038/sj.bjc.6603653 17342094 PMC2360183

[37] Meyskens FL Jr, Surwit E, Moon TE, Childers JM, Davis JR, Dorr RT et al Enhancement of regression of cervical intraepithelial neoplasia II (moderate dysplasia) with topically applied all-trans-retinoic acid: a randomized trial. J Natl Cancer Inst. 1994;86(7):539–43. 10.1093/jnci/86.7.539 8133537

[38] Castle PE, Schiffman M, Wheeler CM, Solomon D. Evidence for frequent regression of cervical intraepithelial neoplasia-grade 2. Obstet Gynecol. 2009;113:18–25. 10.1097/AOG.0b013e31818f5008 19104355 PMC2694845

[39] National Organization for the Provision of Health Services (EOPYY). 2016; http://www.eopyy.gov.gr.

[40] Diaz M, de Sanjose S, Ortendahl J, O’Shea M, Goldie SJ, Bosch FX, et al Cost-effectiveness of human papillomavirus vaccination and screening in Spain Eur J Cancer. 2010;46:2973–85. 10.1016/j.ejca.2010.06.016 20638840

[41] Organization for Economic Co-operation and Development (OECD). OECD Statistics. 2017; http://stats.oecd.org/.

[42] Kim JJ, Burger EA, Regan C, Sy S. Screening for Cervical Cancer in Primary Care. A Decision Analysis for the US Preventive Services Task Force. JAMA. 2018; 320:706–714. 10.1001/jama.2017.19872 30140882 PMC8653579

[43] Lew JB, Simms KT, Smith MA, Hall M, Kang YJ, Xu XM et al Primary HPV testing versus cytology-based cervical screening in women in Australia vaccinated for HPV and unvaccinated: eff ectiveness and economic assessment for the National Cervical Screening Program. Lancet Public Health 2017; 2: e96–107. 10.1016/S2468-2667(17)30007-5 29253402

[44] Holmes J, Hemmett L, Garfield S. The cost-effectiveness of human papillomavirus screening for cervical cancer. A review of recent modelling studies. Eur J Health Econ. 2005;6:30–7. 10.1007/s10198-004-0254-1 15682286

[45] Huh WK, Williams E, Huang J, Bramley T, Poulios N. Cost effectiveness of human papillomavirus-16/18 genotyping in cervical cancer screening. Appl Health Econ Health Policy. 2015;13:95–107. 10.1007/s40258-014-0135-4 25385310 PMC5031721

[46] Kim JJ, Wright T, Goldie SJ. Cost-effectiveness of Human Papillomavirus DNA testing in the United Kingdom, the Netherlands, France and Italy. J Natl Cancer Inst. 2005;97:888–95. 10.1093/jnci/dji162 15956650

[47] Sroczynskia G, Schnell-Indersta P, Mühlbergera N, Langb K, Aidelsburgerb P, Wasemc J, et al Cost-effectiveness of primary HPV screening for cervical cancer in Germany–a decision analysis. Eur J Cancer. 2011;47:1633–46. 10.1016/j.ejca.2011.03.006 21482103

[48] van Rosmalen J, de Kok IMCM, van Ballegooijen M. Cost-effectiveness of cervical cancer screening: cytology versus human papillomavirus DNA testing. BJOG. 2012;119:699–709. 10.1111/j.1471-0528.2011.03228.x 22251259 PMC3489039

[49] Vijayaraghavan A, Efrusy M, Lindeque G, Dreyer G, Santas C. Cost effectiveness of high-risk HPV DNA testing for cervical cancer screening in South Africa. Gynecol Oncol. 2008;112:377–83. 10.1016/j.ygyno.2008.08.030 19081611

[50] Vijayaraghavan A, Efrusy MB, Mayrand MH, Santas CC, Goggin P. Cost-effectiveness of High-risk Human Papillomavirus Testing for Cervical Cancer Screening in Québec. Can J Public Health. 2010;101:220–5. 20737813 10.1007/BF03404377 PMC6973910

[51] Wright T, Huang J, Baker E, Garfield S, Hertz D, Cox JT. The Budget Impact of Cervical Cancer Screening Using HPV Primary Screening. Am J Manag Care. 2016;22:95–105. 26978241

[52] Stoler MH, Schiffman M. Interobserver reproducibility of cervical cytologic and histologic interpretations: realistic estimates from the ASCUS-LSIL Triage Study. JAMA. 2001;21;285:1500–5. 10.1001/jama.285.11.1500 11255427

[53] Dillner J, Rebolj M, Birembaut P, Petry KU, Szarewski A, Munk C, et al Long term predictive values of cytology and human papillomavirus testing in cervical cancer screening: joint European cohort study. BMJ. 2008; 10.1136/bmj.a1754. PMC2658827 18852164

[54] Wright TC, Stoler MH, Behrens CM, Sharma A, Sharma K, Apple R. *Interlaboratory variation in the performance of liquid-based cytology*: *insights from the ATHENA trial*. Int J Cancer, 2014; 134(8): 1835–43. 10.1002/ijc.28514 24122508

[55] Starfield B. Primary care: balancing health needs, services, and technology New York: Oxford University Press; 1998.

[56] World Health Organization. The Financial Crisis and Global Health. WHO 2009; http://www.who.int/mediacentre/events/meetings/2009_financial_crisis_report_en_.pdf. 10.1186/1744-8603-5-17

[57] World Health Organization. Health in times of global economic crisis: Implications for the WHO European Region. 2009; http://www.euro.who.int/__data/assets/pdf_file/0006/66957/RC59_edoc07.pdf.

[58] Sheikh S, Biundo E, Courcier S, Damm O, Launay O, Maes E. A report on the status of vaccination in Europe. Vaccine.2018; 36:4979–4992. 10.1016/j.vaccine.2018.06.044 30037416

[59] Goldhaber-Feibert JD, Stout NK, Salomon JA, Kuntz KM, Goldie SJ. Cost-effectiveness of cervical cancer screening with human papillomavirus DNA testing and HPV-16, 18 vaccination. J Natl Cancer Inst. 2008;100:308–20. 10.1093/jnci/djn019 18314477 PMC3099548

[60] Newhouse JP. Free for all? Lessons from the RAND Health Insurance Experiment. Cambridge, MA: Harvard University Press, 1993.

[61] Kue J, Zukoski A, Keon KL, Thorburn S. Breast and cervical cancer screening: exploring perceptions and barriers with Hmong women and men in Oregon. Journal Ethnicity & Health. 2014;19: 311–327. 23477387 10.1080/13557858.2013.776013 PMC3711956

[62] Trivedi AN, Rakowski W, Ayanian JZ. Effect of Cost Sharing on Screening Mammography in Medicare Health Plans. N Engl J Med 2008; 358:375–383. 10.1056/NEJMsa070929 18216358

[63] Huh WK, Ault KA, Chelmow D, Davey DD, Goulart RA, Garcia FAR. Use of primary high-risk human papillomavirus testing for cervical cancer screening: Interim clinical guidance. Gynecologic Oncology. 2015;136:178–182. 10.1016/j.ygyno.2014.12.022 25579107

[64] World Health Organization. WHO guidelines for screening and treatment of precancerous lesions for cervical cancer prevention. Geneva: WHO press; 2013. 24716265

[65] US Preventive Services Task Force. Cervical Cancer: Screening. 2016;.

[66] Food and Drug Administration. 2016; https://www.fda.gov/newsevents/newsroom/pressannouncements/ucm510251.htm.

[67] UK National Screening Committee. 2016; https://legacyscreening.phe.org.uk/.

[68] von Karsa L., et al (2015). European guidelines for quality assurance in cervical cancer screening. Summary of the supplements on HPV screening and vaccination. Papillomavirus Research, 1: 22–31.

[69] National Institute for Public Health and the Environment. Cervical cancer screening in the Netherlands. 2016; http://www.rivm.nl/en/Documents_and_publications/Common_and_Present/Newsmessages/2014/Cervical_cancer_screening_in_the_Netherlands.

[70] Department of Health, National cervical screening program. 2016; http://www.cancerscreening.gov.au/internet/screening/publishing.nsf/content/future-changes-cervical.

[71] National Screening Unit. 2016; https://www.nsu.govt.nz/health-professionals/national-cervical-screening-programme/primary-hpv-screening.

[72] Gültekin M, Akgül B. HPV screening in Islamic countries. The Lancet. 2017;17:368. 10.1016/S1473-3099(17)30126-3 28346175

[73] Rodriguez RC. Cervical Cancer Control in Latin America and the Caribbean: Roundtable Policy Brief. 2016; wwwargentin.org/sites/default/files/resources/UICC%20LAC%20CaCx%20Policy%20Brief%20(Eng)%20Final.pdf

[74] Ronco G, Giorgi Rossi P, Giubilato P, Del Mistro A, Zappa M, Carozzi F, et al A first survey of HPV-based screening in routine cervical cancer screening in Italy. Epidemiol Prev. 2015;39:77–83. 26405779

