# Supplementary material for: Economic evaluation of HPV DNA test as primary screening method for cervical cancer: A health policy discussion in Greece
Source: PLoS One. 2019 Dec 12;14(12):e0226335. doi: 10.1371/journal.pone.0226335 (PMC6907825; doi:10.1371/journal.pone.0226335)
Supplement: S1 Table — (DOCX) [file pone.0226335.s001.docx]

**S1 Table. All model parameters and sensitivity analyses range**

| Parameters | Base Case Value | Lower Value | Upper Value |
| --- | --- | --- | --- |
| Compliance parameters |  |  |  |
| Attedance rate for management algorithms | 30,3% | 20% | 80% |
| Compliance rate for cervical cancer screening | 30,3% | 20% | 80% |
| Cost parameters (±20%) | | |  |
| Office visit (routine/repeat screening) cost | 10,00 € | 8,00 € | 12,00 € |
| Office visit (diagnostic follow up) cost | 10,00 € | 8,00 € | 12,00 € |
| Cytology test (liquid based) cost | 13,32 € | 10,66 € | 15,98 € |
| Cytology test (conventional) cost | 13,32 € | 10,66 € | 15,98 € |
| Cytology test additional cost of abnormal test cost | 13,32 € | 10,66 € | 15,98 € |
| HPV DNA testing cost | 80,00 € | 64,00 € | 96,00 € |
| Linear array HPV genotyping test cost | 80,00 € | 64,00 € | 96,00 € |
| Colposcopy plus biopsy cost | 38,74 € | 30,99 € | 46,49 € |
| Treatment for CIN 2+ cost | 1.533,02 € | 1.226,41 € | 1.839,62 € |
| Treatment of invasive cervical cancer cost | 20.572,60 € | 16.458,08 € | 24.687,12 € |
| Discount rate for cost | 3,5% | 0% | 6% |
| Clinical parameters * |  |  |  |
| Discount rate for clinical outcomes | 3,5% | 0% | 6% |
| *Cytology (threshold=ASCUS)* |  |  |  |
| Sensitivity of cytology for CIN 2+ (ASCUS) | 53,70% | 37% | 69,30% |
| Sensitivity of cytology for CIN 3+ (ASCUS) | 64,30% | 35% | 87,20% |
| Sensitivity of cytology for CC (ASCUS) | 64,30% | 35% | 87,20% |
| Specificity of cytology (ASCUS) | 96,80% | 96% | 97,40% |
| *Cytology (threshold = LSIL)* |  |  |  |
| Sensitivity of cytology for CIN 2+ (LSIL) | 41,50% | 26,30% | 57,90% |
| Sensitivity of cytology for CIN 3+ (LSIL) | 57,10% | 28,90% | 82,30% |
| Sensitivity of cytology for CC (LSIL) | 57,10% | 28,90% | 82,30% |
| Specificity of cytology for CIN2+ (LSIL) | 98,80% | 98,40% | 99,10% |
| *Cytology (threshold = HSIL)* |  |  |  |
| Sensitivity of cytology for CIN 2+ (HSIL) | 17,70% | 8,50% | 31,30% |
| Sensitivity of cytology for CIN 3+ (HSIL) | 21,40% | 7,60% | 47,60% |
| Sensitivity of cytology for CC (HSIL) | 21,40% | 7,60% | 47,60% |
| Specificity of cytology for CIN 2+ (HSIL) | 99,80% | 99,60% | 99,90% |
| *HPV testing* |  |  |  |
| Sensitivity of pooled hrHPV testing for CIN2 | 100% | 91,40% | 100% |
| Sensitivity of pooled hrHPV testing for CIN3 | 100% | 76,80% | 100% |
| Sensitivity of pooled hrHPV testing for CC | 100% | 76,80% | 100% |
| Specificity of pooled hrHPV testing for CIN2+ | 100,00% | 89,30% | 91,20% |
| Sensitivity of HPV with genotyping for CIN2 | 58,50% | 42,10% | 73,70% |
| Sensitivity of HPV with genotyping for CIN3 | 78,60% | 49,20% | 95,30% |
| Sensitivity HPV with genotyping for CC | 78,60% | 49,20% | 95,30% |
| Specificity of HPV with genotyping for CIN2+ | 97,50% | 96,90% | 98% |
| Epidemiological parameters (±10%) |  |  |  |
| Prevalence of 14hrHPV | 12,70% | 11,43% | 13,97% |
| Prevalence of HPV16 and/or 18 | 3,90% | 3,51% | 4,29% |
| Prevalence of CIN1 | 2,10% | 1,89% | 2,31% |
| Prevalence of CIN2 | 0,70% | 0,63% | 0,77% |
| Prevalence of CIN3 | 0,40% | 0,36% | 0,44% |
| Prevalence of invasive cervical cancer | 0,05% | 0,05% | 0,06% |
| Natural History Parameters (±5%) |  |  |  |
| Well to hrHPV infection | 4,24% | 4,03% | 4,45% |
| *Progression from hrHPV (12 types)* |  |  |  |
| Progression from hrHPV (12 types) to CIN1 | 8,06% | 7,66% | 8,46% |
| Progression from hrHPV (12 types) to CIN2 | 0,05% | 0,05% | 0,05% |
| Progression from hrHPV (12 types) to CIN3 | 0,13% | 0,12% | 0,14% |
| *Progression from hrHPV 16/18* |  |  |  |
| Progression from hrHPV 16/18 to CIN1 | 9,87% | 9,38% | 10,36% |
| Progression from hrHPV 16/18 to CIN2 | 0,60% | 0,57% | 0,63% |
| Progression from hrHPV 16/18 to CIN3 | 1,50% | 1,43% | 1,58% |
| *Progression from CIN1* |  |  |  |
| Progression from CIN1 to CIN2 | 3,25% | 3,09% | 3,41% |
| Progression from CIN1 to CIN3 | 0,87% | 0,83% | 0,91% |
| *Progression from CIN2* |  |  |  |
| Progression from CIN2 to CIN3 | 4,17% | 3,96% | 4,38% |
| Progression from CIN2 to Invasive Cervical Cancer | 0,00% | 0,00% | 0,00% |
| *Progression from CIN3* |  |  |  |
| Progression from CIN3 to Invasive Cervical Cancer | 1,13% | 1,07% | 1,19% |
| Annual mortality rate for cervical cancer | 0,64% | 0,61% | 0,67% |
| *Regression from hrHPV (12 types)* |  |  |  |
| Regression from hrHPV (12 types) with NORMAL smear to well | 58,60% | 55,67% | 61,53% |
| Regression from hrHPV (12 types) with BORDERLINE/MILD smear to well | 45,55% | 43,27% | 47,83% |
| *Regression from hrHPV 16/18* |  |  |  |
| Regression from hrHPV 16/18 with NORMAL smear to well | 43,76% | 41,57% | 45,95% |
| Regression from hrHPV 16/18 with BORDERLINE/MILD smear to well | 21,83% | 20,74% | 22,92% |
| *Regression from CIN1* |  |  |  |
| Regression from CIN1 to well | 21,16% | 20,10% | 22,22% |
| Regression from CIN1 to hrHPV | 2,35% | 2,23% | 2,47% |
| *Regression from CIN2* |  |  |  |
| Regression from CIN2 to well | 9,39% | 8,92% | 9,86% |
| Regression from CIN2 to CIN1 | 9,39% | 8,92% | 9,86% |
| *Regression from CIN3* |  |  |  |
| Regression from CIN3 to well | 9,39% | 8,92% | 9,86% |
| Regression from CIN3 to CIN1 | 1,59% | 1,51% | 1,67% |

* The sensitivity analyses range (lower and upper values) was drawn from Agorastos et al. (2015)
