# Supplementary material for: Economic evaluation of HPV DNA test as primary screening method for cervical cancer: A health policy discussion in Greece
Source: PLoS One. 2019 Dec 12;14(12):e0226335. doi: 10.1371/journal.pone.0226335 (PMC6907825; doi:10.1371/journal.pone.0226335)

**S2 Fig. One way sensitivity analysis results of 5-year HPV testing with simultaneous 16/18 genotyping versus annual cytology (all parameters results ranked according to their impact)**

**A) Cost**

**
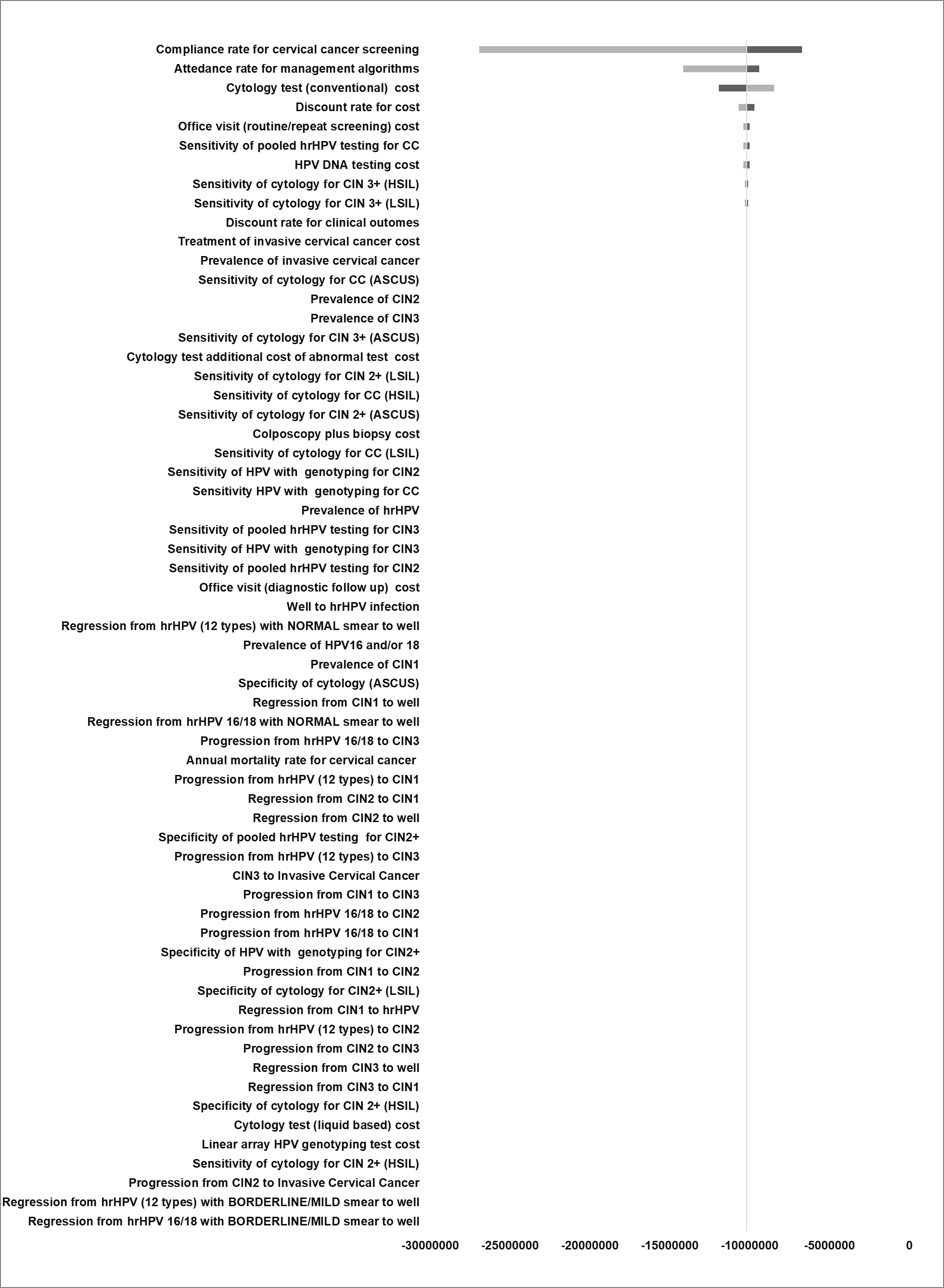
**

**B) Deaths
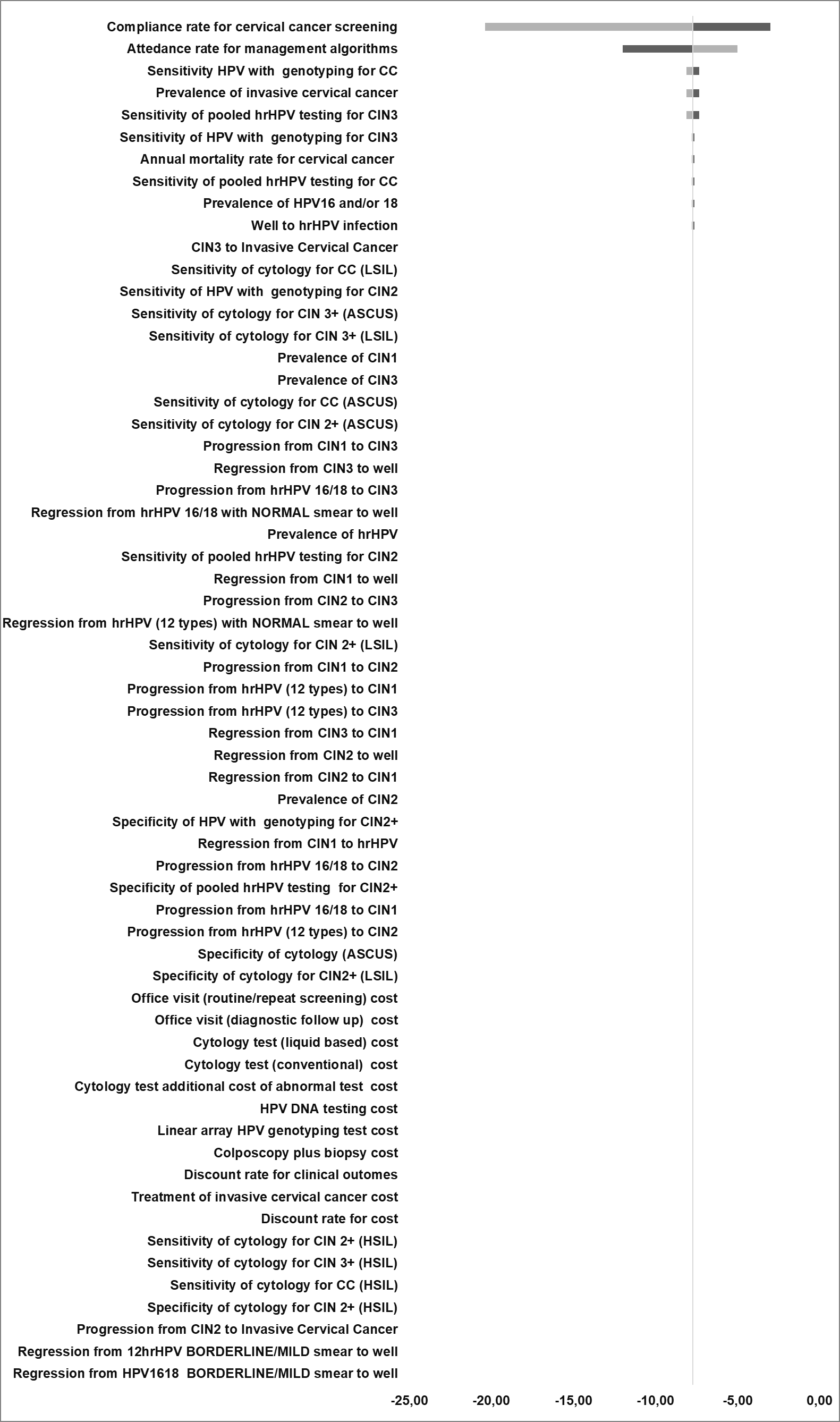
**

**C) Incidence**
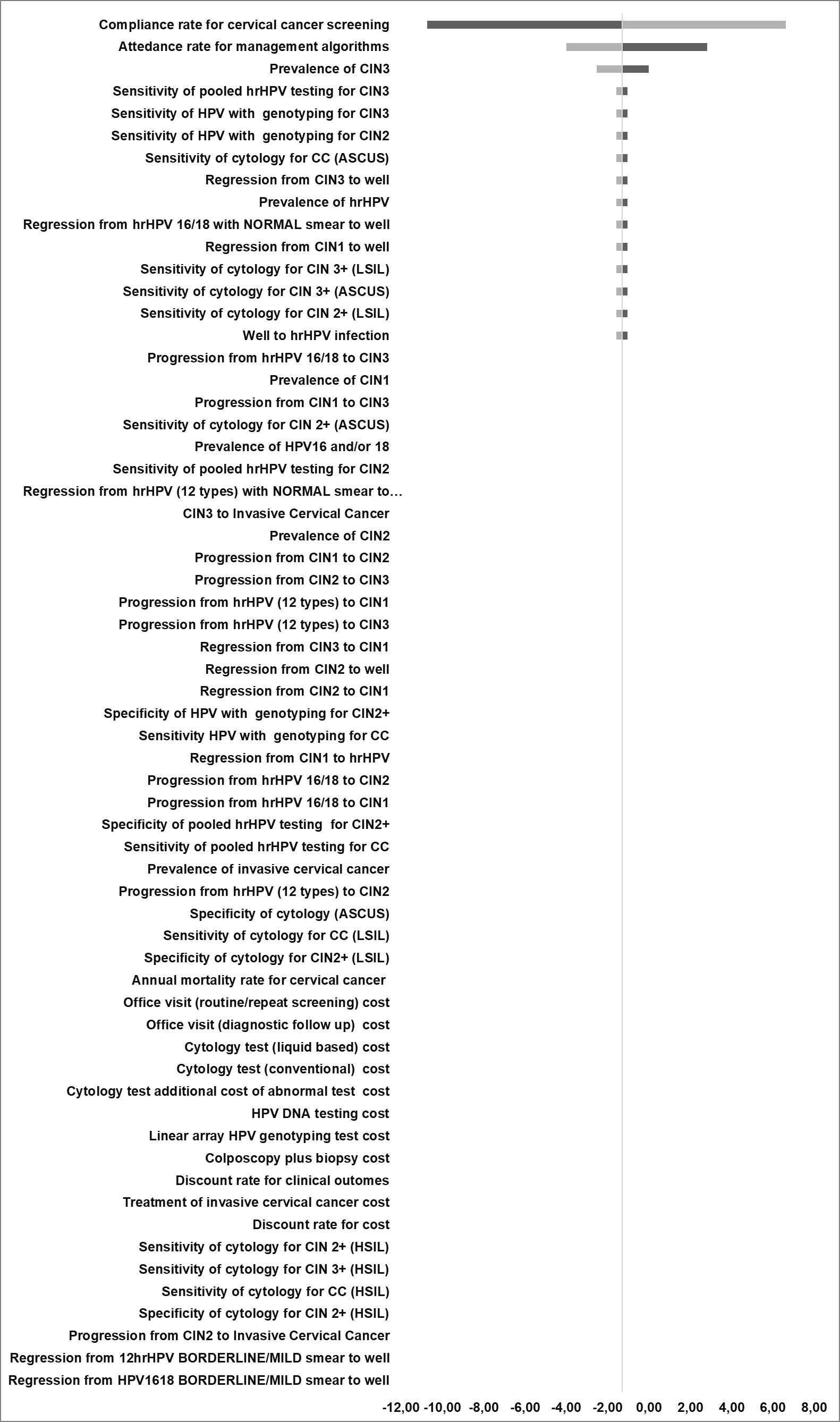

Supplement: S2 Fig — (DOCX) [file pone.0226335.s003.docx]
